# Comparative cross-linking and mass spectrometry of an intact F-type ATPase suggest a role for phosphorylation

**DOI:** 10.1038/ncomms2985

**Published:** 2013-06-12

**Authors:** Carla Schmidt, Min Zhou, Hazel Marriott, Nina Morgner, Argyris Politis, Carol V. Robinson

**Affiliations:** 1 Department of Chemistry, Physical and Theoretical Chemistry Laboratory, University of Oxford, Oxford OX1 3QZ, UK

## Abstract

F-type ATPases are highly conserved enzymes used primarily for the synthesis of ATP. Here we apply mass spectrometry to the F_1_F_O_-ATPase, isolated from spinach chloroplasts, and uncover multiple modifications in soluble and membrane subunits. Mass spectra of the intact ATPase define a stable lipid ‘plug’ in the F_O_ complex and reveal the stoichiometry of nucleotide binding in the F_1_ head. Comparing complexes formed in solution from an untreated ATPase with one incubated with a phosphatase reveals that the dephosphorylated enzyme has reduced nucleotide occupancy and decreased stability. By contrasting chemical cross-linking of untreated and dephosphorylated forms we show that cross-links are retained between the head and base, but are significantly reduced in the head, stators and stalk. Conformational changes at the catalytic interface, evidenced by changes in cross-linking, provide a rationale for reduced nucleotide occupancy and highlight a role for phosphorylation in regulating nucleotide binding and stability of the chloroplast ATPase.

## Abstract (web summary)

Rotary ATPases are membrane-embedded motors that produce or consume ATP and control pH within cells. Schmidt *et al.* use mass spectrometry to characterize the intact chloroplast ATPase from spinach and, using comparative cross-linking, show that phosphorylation affects stability and nucleotide occupancy.

## Graphical abstract

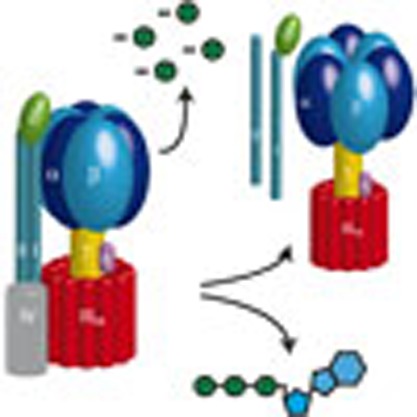

ATP synthases are membrane-embedded rotary motors that produce or consume ATP and control the pH within cells^1^. All rotary ATPases (F-, V- and A-type) have a conserved architecture based on a water-soluble F_1_, V_1_ or A_1_ domain, and a membrane-bound proton-translocating F_O_, V_O_ or A_O_ domain. As yet, there are no high-resolution structures of intact rotary ATPases, although considerable insight has been amassed by docking high-resolution structures into electron microscopy density maps^2^,^3^. These studies are providing detailed information of the subunit interactions, function and motion of V- and F-type ATPases from thermophilic bacteria, yeast and mitochondria^2^,^4^,^5^.

The ATP synthase that operates in the thylakoid membranes of chloroplasts (the CF_1_F_o_–ATPase (cATPase)) is energized by a proton gradient (ΔpH) and shares structural components with other F-type ATPases. F_1_ consists of (α_3_β_3_γδε) and two peripheral subunits I and II forming the stator stalk. F_O_ is composed of 14 copies of subunit III in a ring and 1 copy of subunit IV. Interactions are established between subunits δ, II and IV, forming the peripheral stalk, and the α_3_β_3_ soluble head, attached to subunit I^6^. The ε-subunit and part of the γ-subunit comprise the central stalk connected to the (III)_14_ subunits and are responsible for rotation under the pH gradient. In line with other F-type ATPases, there are six possible nucleotide-binding sites (three catalytic and three regulatory) at αβ subunit interfaces of which five are predicted to be occupied^7^. Interestingly, however, after isolation the cATPase was found to contain only one endogenous ADP at one of the catalytic sites and two endogenous ATP molecules at non-catalytic sites^8^. In the X-ray structure of the F_1_ complex from cATPase all α and β subunits adopt a closed conformation and appear to contain no bound adenine nucleotides^9^.

Regulation of cATPase occurs via a variety of different mechanisms, including inhibition by tight binding of ADP and Mg^2+^ (10), in common with other F-type ATPases. Specific to photosynthetic organisms, the F_1_ subunits γ and ε are also thought to have critical roles in ATPase regulation in response to light and dark adaptation, with γ likely having the dominant role^11^.

Recently, we have shown that two V-type ATPases from bacteria could be projected into the gas phase of a mass spectrometer and, through excitation, could be released intact from detergent micelles. This enabled us to measure subunit and lipid-binding stoichiometries, as well as to probe the effects of nucleotide binding on subunit interfaces^12^.

Here we target the intact chloroplast ATP synthase from *Spinacia oleracea* purified in-house. Specifically, we investigate the influence of post-translational modifications (PTMs) on the stability of subunit interfaces and nucleotide binding. By comparative cross-linking, we probe the relationship between changes in subunit interactions and loss of nucleotides. Linking these two events reveals molecular details of the conformational changes that take place in the head of the ATPase, highlighting a regulatory role for phosphorylation via mediation of interactions between the head and the ε-subunit, the stators and the α/β interface.

## Results

### Characterization of protein subunits

Chloroplast ATP synthase was isolated from spinach leaves (Supplementary Methods and Supplementary Fig. S1) and the nine anticipated protein subunits α, β, γ, δ, ε, I, II, III and IV were confirmed by peptide sequencing following in-gel digestion (Supplementary Table S1). Masses of the intact protein subunits ±2.5 Da) were then determined using liquid chromatography–mass spectrometry (LC–MS), enabling precise masses to be assigned to each subunit (Supplementary Figs S2 and S3, and Supplementary Table S2). Protein subunits α, β and ε show excellent agreement between theoretical and experimentally determined masses. Small differences in mass are attributed to PTMs, such as loss of amino-terminal methionine and/or N-terminal acetylation, as well as multiple oxidations observed for subunit III (Supplementary Fig. S3). Protein subunits γ, δ, I, II and IV have significantly lower masses than the theoretical masses calculated from the database due to loss of N-terminal presequences upon import into the chloroplast (subunits δ, γ and II)^13^,^14^,^15^,^16^ or by N-terminal truncation (subunits I and IV)^13^,^17^.

### Characterization of the ‘lipid plug’

**Figure 1:**
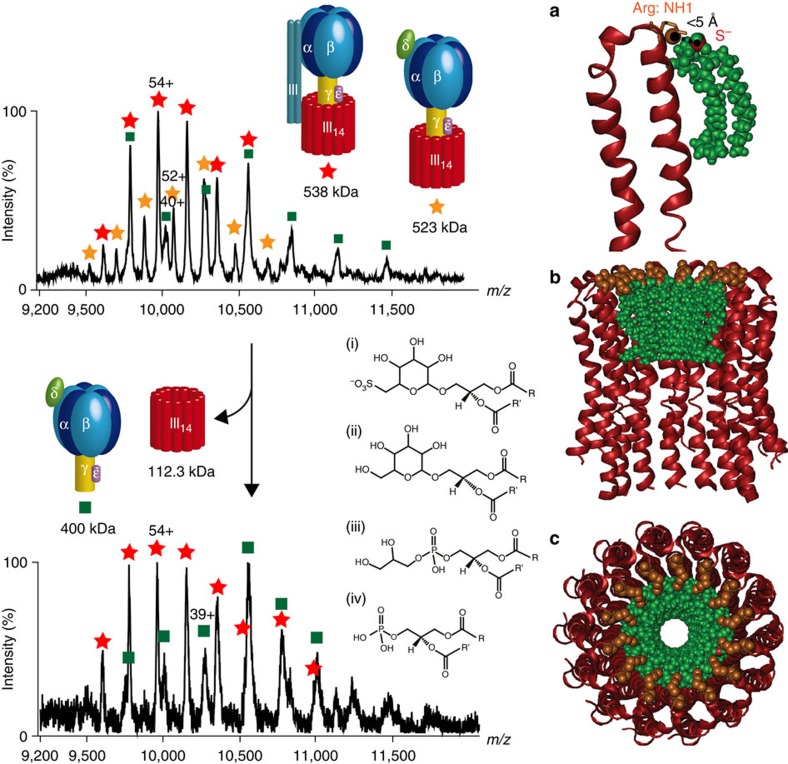
F_1_F_O_ complexes allow identification of the lipid plug and define the protein:lipid stoichiometry within the membrane ring. F_1_F_O_ complexes of the cATPase formed in solution were assigned (upper spectrum), but were found to be unstable during incubation at 37 °C. The mass difference between the 523-kDa complex (orange star) and the complex formed following incubation (400 kDa, lower spectrum, green square) is attributed to loss of the membrane ring with bound lipids. Different lipids were identified with different isoforms: sulpholipid (i), glycolipid (ii) and two phospholipids (iii and iv). The average mass of these lipids and the mass of the lipid plug (~10.5 kDa) reveal a protein:lipid stoichiometry of 1:1. The most abundant negatively charged sulpholipid was docked onto subunit III at its positively charged arginine residue; side view (**a**) side view of the III_14_ ring with docked lipids (**b**) and the inner diameter of the membrane ring is reduced by bound lipids, top view (**c**). The spectra shown represent an experiment from at least three replicates.

Using a procedure described previously^18^,^19^, we recorded a mass spectrum for the intact cATPase, introduced in a *n*-dodecyl-β-D-maltopyranoside detergent micelle (Fig. 1). By employing a mass spectrometer modified for transmission of high-mass complexes^20^ and by following activation in the gas phase, the detergent micelle was removed while non-covalent interactions were preserved. At least three complexes were observed with masses of 400, 523 and 538 kDa. Although the 400 kDa species agrees well with the mass of the intact F_1_ domain (α_3_β_3_γδε), the other two complexes were 120 kDa greater than anticipated from summing the subunits of the F_1_ or F_O_ complexes alone. This implies that these complexes consist of both membrane and soluble subunits. However, it was not possible to assign these complexes based on subunit masses alone implicating the presence of lipid components, analogous to previous observations^12^. To investigate this, we incubated the cATPase-containing solution at 37 °C for 1 h and recorded a spectrum of the subcomplexes that persist under these conditions. We noted that the 523 kDa complex disassembled readily, whereas the 538 kDa complex was stable under these conditions. The difference in mass between the 523 and 400 kDa complex, which was formed following incubation at 37 °C, was 123 kDa, which is close to the mass anticipated for the membrane ring (112.3 kDa) together with a lipid plug of 10.5 kDa.

To identify the bound lipids within the membrane ring, we digested the intact cATPase and subjected the peptide/lipid mixture to LC–MS/MS analysis. We identified four different classes of lipids: two phospholipids, one glycolipid and one sulpholipid (Fig. 1, Supplementary Fig. S4 and Supplementary Table S3). The four lipids were identified as different isomers, with diverse fatty acid side chains and with a range of intensities (Supplementary Table S3). The average mass of the lipids was calculated as 750 Da. This mass, together with that of the lipid plug (10.5 kDa), and the protein stoichiometry of III_14_ in the membrane ring^21^ equates to a membrane protein III:lipid stoichiometry of 1:1, implying stoichiometric binding within the membrane ring. Considering the negatively charged sulpholipid, the most abundant lipid identified, and the location of the positively charged arginine, at the top of the ring on the inner face of the III_14_ ring, we propose a model for the likely binding orientation for this lipid plug. Binding of the sulpholipid reduces the central orifice of the membrane ring as seen previously for V-type ATPases^12^. Interestingly, the proportion of the space occupied by lipids in the cavity is much greater for the F-type ATPases, consistent with the smaller diameter of the γ-subunit compared with the C subunit in V-type ATPases (Supplementary Fig. S5).

### Dissociation pathways of the cATPase

**Figure 2:**
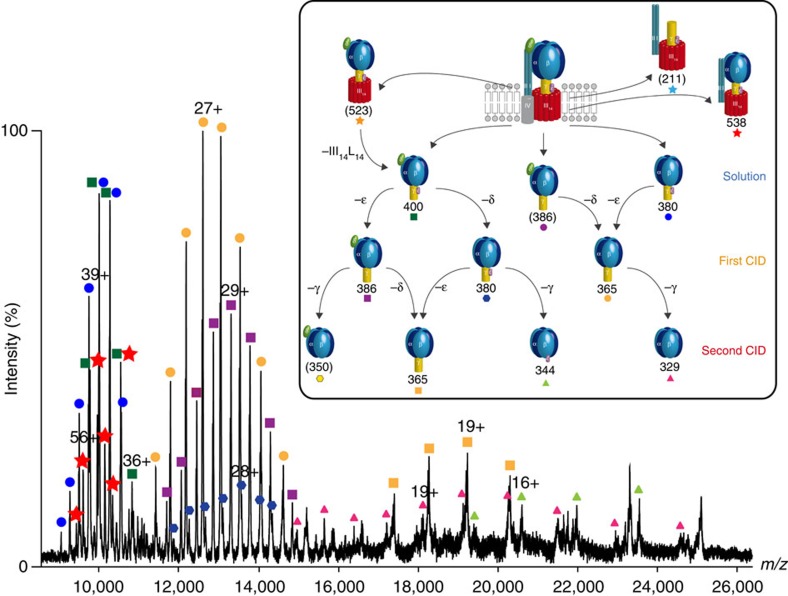
A mass spectrum of solution and gas-phase dissociation products of the cATPase. At high activation energy, micelle-stripped complexes are observed, with those between 9,000 and 11,000 *m/z* assigned to complexes formed in solution. First and second generation CID products are formed by the loss of one or two subunits observed between 12,000 and 14,000 *m/z*, and 16,000 and 25,000 *m/z*, respectively. The spectrum represents a typical cATPase mass spectrum from at least three replicates. A schematic representation of the solution and gas-phase dissociation pathways is shown (inset). Brackets denote complexes identified in mass spectra recorded under different conditions.

Having defined the lipid plug and accurate masses of all subunits, it was possible to assign all subcomplexes using an assignment strategy reported previously (Massign)^22^ (Fig. 2, Supplementary Figs S6 and S7, and Supplementary Table S4). From these assignments, together with the overall charge of the complex, we could deduce that the largest subcomplexes, formed in solution, preserve interactions between the head and the base (538 kDa) even in the absence of the stator subunits (523 kDa). Interestingly, subunits I and II are only connected to complexes when the membrane ring is present (Supplementary Fig. S7). The first and second generation gas-phase dissociation products of these complexes show that they have lost stator subunits I and II, as well as subunits δ, ε and γ (Fig. 2 and Supplementary Fig. S6). The membrane ring subunit III and its multimers, δ, I, II and IV, are observed at low *m/z* values (Supplementary Fig. S8). We attribute the absence of subunit IV in all complexes to its hydrophobic interactions with the membrane ring and the absence of the lateral force provided by the membrane. Of interest is the complex formed from the soluble head in which the ε subunit is attached directly in the absence of γ (344 kDa), as ε must interact directly with the head rather than being mediated through γ. This direct interaction, not reported previously, is likely the result of a regulation mechanism in which a population of cATPases is trapped in an inactive state.

To confirm the assignment of the subcomplexes, we carried out in-solution disruption by manipulating the ionic strength or pH (Supplementary Figs S9 and S10). Under basic pH conditions several subcomplexes were formed, as both ε and δ subunits dissociate readily to yield a complex with the base and head associated with subunits I, II and γ. Surprisingly, under high ionic strength and at pH 5.0, no further subcomplexes were generated, implying that this 538 kDa complex is stable in solution without δ and IV. We conclude that in the absence of F_O_, and the stator subunits (I and II), complexes of the F_1_ head dissociate readily. By contrast, the 538-kDa F_1_F_O_ complex is stable, its strength conferred by binding of subunits I and II.

### Subunit interactions at the molecular level

To investigate subunit interactions at the molecular level we applied chemical cross-linking to the cATPase. Using the cross-linking reagent BS3, we identified 958 hits after database searching using the defined mass difference between the two cross-linkers and the sequences of all proteins. We validated 869 cross-links after manual inspection of the mass spectra giving a false discovery rate of 9.29%, corresponding to 105 different cross-links (Supplementary Table S5 and Supplementary Fig. S11). Disregarding intraprotein cross-links, 32 unique interprotein cross-links remain within the cATPase. Projecting our cross-links onto the available X-ray crystal structure of the F_1_ α_3_β_3_ head (PDB 1FX0), we found five cross-links within the region defined crystallographically (all ≤35 Å). BS3 has a distance constraint of 11.4 Å adding to this 6.5 Å for each cross-linked lysine side chain (≈24.4 Å) and allowing for conformational dynamics (≈35 Å) (Supplementary Fig. S12). Interestingly, many of the flexible termini of the soluble subunits absent in the X-ray structure, for example, the carboxy-terminal residue K498 of the β-subunit, cross-linked to multiple protein subunits.

**Figure 3:**
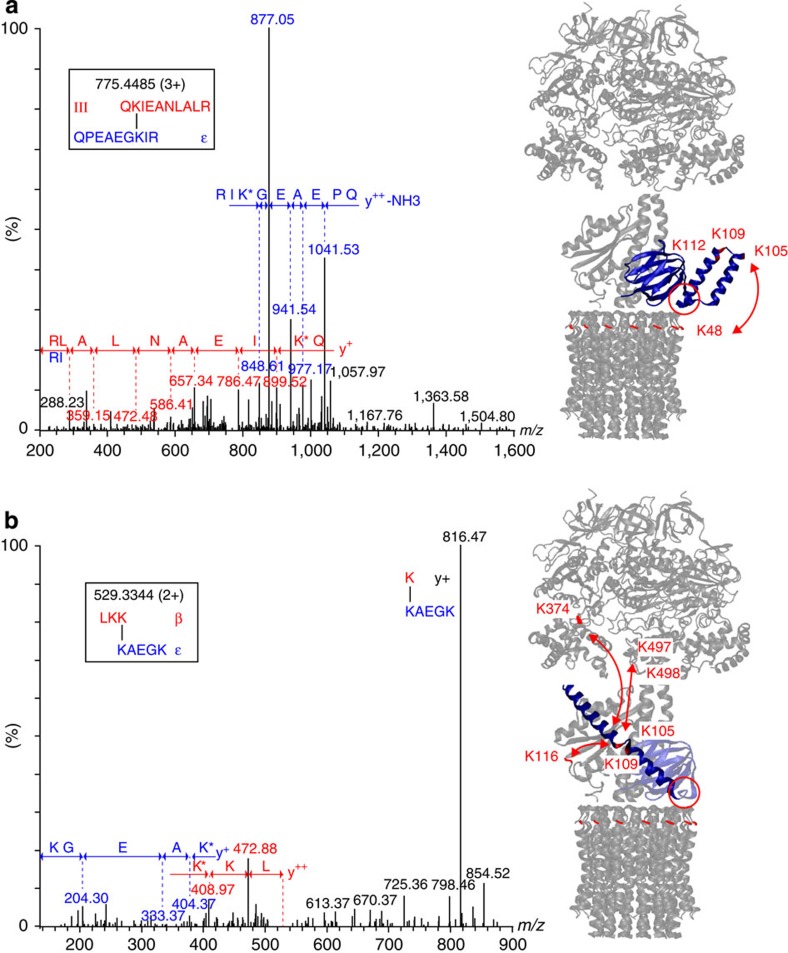
Cross-linking of the cATPase reveals two different conformations of subunit ε. Representative mass spectra of two cross-linked peptides derived from ε, one cross-linked to subunit III in the membrane ring (**a**) and the other to β in the F_1_ head (**b**). These two different cross-links are consistent with compact and extended forms of subunit ε. A key phosphorylation site at the interface of the N- and C-terminal domains of the ε-subunit is shown (red circle) and cross-linked residues are shown (red). The following cross-links were identified, upper panel: ε-109, ε-105 and ε-112, all cross-link with subunit III-48; and lower panel: ε-109 with γ-116 and ε-105 with β-498 and β-497, and ε-105 with α-374.

For subunits without atomic structures, we constructed homology models using automated approaches, to implement our cross-linking information in a structural context. Models for ε, δ and γ were generated using MODELLER^23^ and subsequently scored (Supplementary Table S6). Identified cross-links were within the cut-off distance for models of ε and γ, validating their modelled structures (Supplementary Fig. S13). For the δ subunit, only a partial model was obtained, which was included for completeness. A single cross-link was located in the flexible, solvent accessible part of δ, whereas γ was highly cross-linked (Supplementary Fig. S13C,D) in line with its central position, in contact with four different subunits, and highlighting its solvent accessibility. Of interest were cross-links between the ε subunit and the membrane ring, as well as γ, α and β. Given the size and anticipated fold of ε to make contact with the membrane ring and the head, ε would have to be in closed and extended conformations, respectively (Fig. 3). A population of cATPases must therefore have extended the loop region such that the C-terminal helix of ε contacts the α_3_β_3_ head. Cross-linking of ε in two different directions is consistent with subcomplexes formed in the absence of cross-linker, validating our proposal that cATPases in this preparation are in both active and inactive forms.

**Figure 4:**
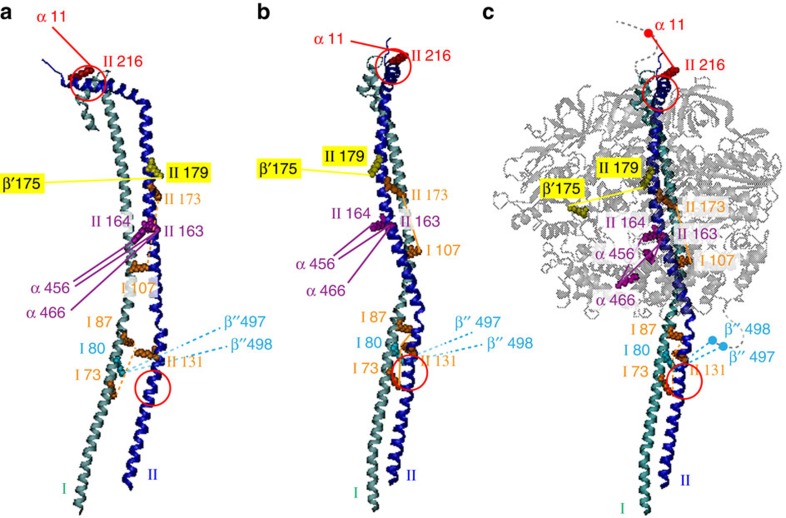
Cross-links define interactions between the two peripheral stalks and locate their binding to the head. Side view of the two stalk subunits (**a**), the front view (**b**) and their location on the head is shown (**c**). Three intersubunit cross-links define the interactions between lysines in subunits I and II (orange). Interactions with α and β subunits are labelled with the respective protein name and the residue number. Interactions in the front and behind the plan are denoted with solid and dashed lines, respectively. Flexible loops of the α and β subunits absent in the X-ray structure are added manually (gray, dashed lines). The two phosphorylation sites in subunit II (S122 and S211) are indicated (red circles).

Cross-linking also defines multiple interactions between subunits I and II, and with α and β locating their binding on the F_1_ head (Fig. 4). For subunits I and II, predominantly helical structures have been predicted by the secondary structure prediction tool (PSIPRED)^24^ (Supplementary Figs S14 and S15, and Supplementary Table S7). Three intersubunit cross-links define the interactions between lysines in I and II. For connections to the head, subunit I has only two connections in the lower part of the structure, with the flexible loop of the β-subunit. By contrast, five cross-links are assigned to subunits II, and α and β, implying that II is accessible to the cross-linking reagent, protecting I such that I likely connects directly to the head.

### Phosphorylation-dependent stability and nucleotide binding

**Figure 5:**
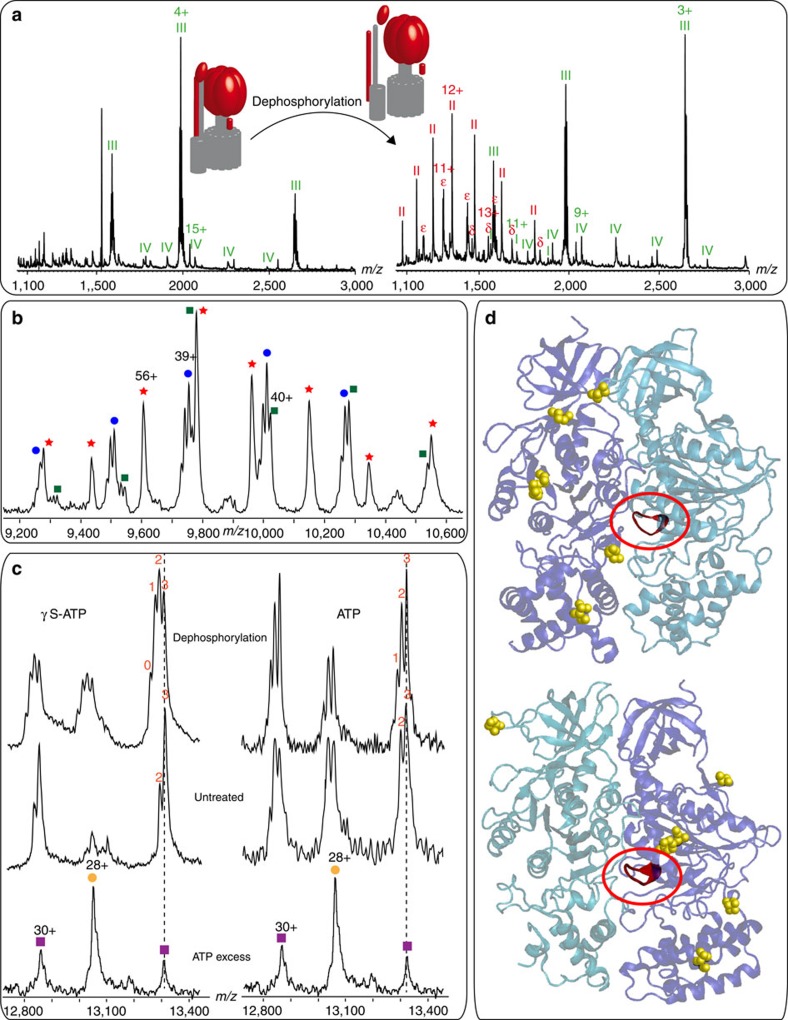
Dephosphorylation effects nucleotide occupancy of the cATPase. (**a**) Mass spectra showing the low *m/z* region of the cATPase in its phosphorylated (untreated) (left-hand side) and its dephosphorylated form (right-hand side). Phosphosites have been identified in subunits α, β, δ, ε and II (red). Incubation with a phosphatase leads to release of additional subunits, assigned to phosphorylated proteins. (**b**) Complexes of the cATPase formed in solution following dephosphorylation. Peaks are labelled with the same symbols as used in Figs 1 and 2. The peaks reveal splitting with a mass difference that can be attributed to nucleotides. (**c**) The cATPase was purified with ATP or the non-hydrolysable analogue γS-ATP. When incubated with an excess of ATP (tenfold), three nucleotides are bound (lower spectrum). Both purifications (with ATP and γS-ATP) showed populations with two and three nucleotides bound in the untreated form at an occupancy ratio of 1:1 and 1:2, respectively (middle trace). Dephosphorylated cATPases showed additional peaks corresponding to populations with one or zero nucleotides bound (upper spectrum). The occupancy ratios for these populations are 1:2:4:5 and 2:4:5:4 for ATP and γS-ATP purifications, respectively. (**d**) The structures of the α/β and β/α interfaces are shown (upper and lower panels respectively; pdb coordinates 1FX0). The catalytic (upper panel) and the non-catalytic (regulatory, lower panel) nucleotide-binding sites (P-loops) are highlighted in red. The phosphosites in the α and β subunits are shown (yellow space filling). Spectra shown represent one experiment from at least three replicates.

As subunit interactions are often affected by phosphorylation, we used a titanium dioxide enrichment strategy^25^ and identified phosphosites on subunits α (S127, Y196, S369, T442), β (S8, T54), δ (S97), ε (S13) and II (S122, S211) (Supplementary Table S8 and Supplementary Fig. S16). To determine the effects of these phosphorylation sites on the stability of the various subcomplexes, we incubated the cATPase with a phosphatase. At the low *m/z* region of the mass spectrum, subunits I, II, ε and δ are observed consistent with a reduction in subunit interactions upon dephosphorylation (Fig. 5a). Stable complexes containing F_O_F_1_ (538 kDa) and those containing only F_1_ components (440 and 380 kDa) survive up to 2 h of incubation with the phosphatase after which time all complexes have dissociated (Fig. 5b). Loss of I and II is rationalized, as the phosphatase has access to the peripheral subunit II, its release likely destabilizing subunit I. Interestingly for the 400 kDa complex, formed via loss of the lipid plug and the membrane ring, the resolution of the mass spectral peaks increases under these conditions, revealing peak splitting corresponding to different numbers of nucleotides binding within the soluble head.

To investigate nucleotide binding we recorded mass spectra of cATPase purified with ATP at 2 mM concentration, and subsequently increased this to 10 mM. A maximum of three nucleotides bind stably within the complex under these conditions (Fig. 5c, lower panel). Comparing this spectrum with two preparations of cATPase isolated from preparations containing ATP or the non-hydrolysable analogue of ATP γS-ATP (2 mM concentration of nucleotide) showed, on average, maximal occupancy of three or two nucleotide-binding sites. We then dephosphorylated the enzymes from the different preparations and compared the untreated enzyme with the one that has undergone dephosphorylation. We observed broader population of nucleotide-bound states for dephosphorylated forms for both preparations (0–3) versus for the untreated enzyme (2–3) (Fig. 5c). Dephosphorylation reduces the nucleotide-binding site occupancy for cATPases isolated from preparations containing either ATP or γS-ATP, ruling out the possibility that loss of nucleotide arises from dephosphorylation of ATP. Considering the location of the phosphosites within the structure of the soluble head, five are equidistant along one face of α/β interface (Fig. 5d). Their location, therefore, suggests a mechanism whereby dephosphorylation promotes a weakening of the subunit interfaces and is coupled to facile release of nucleotides from the soluble head.

### Phosphorylation-dependent conformational changes

**Figure 6:**
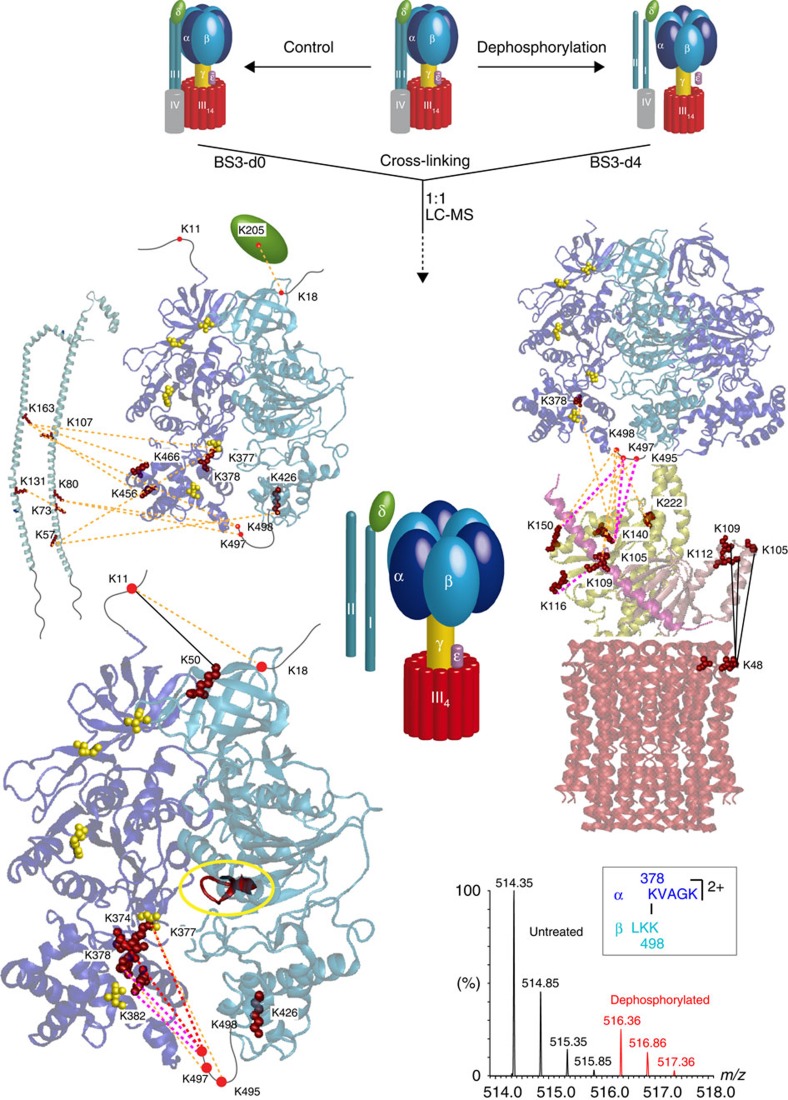
Cross-linking allows direct comparison of the untreated cATPase with the dephosphorylated form. Interactions determined by a comparative cross-linking procedure (top schematic). No change in the extent of cross-linking between the two forms of the enzyme is represented with a solid black line. A reduction in cross-linking in the dephosphoylated form is shown by a two- to fivefold reduction (orange), 5–10 (magenta), >10 (red). Subunit δ is shown as a schematic. Phosphosites are shown in yellow. Changes in interactions are observed between the α/β interfaces and their interactions with subunits I, II, γ, δ and the extended structure of ε. Interactions between ε and the membrane ring are not affected (black).

Having established that both nucleotide occupancy and subunit interactions are compromised when the enzyme is dephosphorylated, we carried out a comparative chemical cross-linking study, comparing the desphosphorylated form of the enzyme with the untreated cATPase. Our strategy involved first cross-linking the untreated enzyme with BS3 and the dephosphorylated enzyme with a deuterium-labelled cross-linker (BS3-d_4_), mixing, digestion, separation and MS (Supplementary Fig. S17). Comparing the intensities of cross-linked peptides in spectra, we found that the majority of intrasubunit cross-links were unchanged (Supplementary Table S9). Major differences occurred in the intensity of 28 of the 32 interprotein cross-links, the intensity of cross-linked peptides in the dephosphorylated enzyme being significantly decreased (three- to tenfold reduction; Supplementary Table S9). An α:β cross-link from K378 to K498 in which the intensity of the cross-link is decreased for the dephosphorylated form of the enzyme approximately fivefold is shown (Fig. 6). Interestingly, four interprotein cross-links do not change in response to dephosphorylation, significantly between ε and the membrane ring and between α and β subunits at the top of the head, implying that rather than overall stability of the complex conformational changes perturb subunit interfaces (Fig. 6). Considering those cross-links that change significantly in response to dephosphorylation, many are between subunits I and II and at the α/β interface. Given the location of phosphorylation sites on subunit II, at the top of the head and the unstructured C terminus of β, dephosphorylation perturbs these interactions, providing a rational for loss of subunits I and II in solution during incubation with the phosphatase (Fig. 5a). Interestingly, cross-links between ε and the membrane ring are retained, whereas those with ε and the soluble head are reduced, presumably due to disruption of the soluble head while the membrane ring, which is not phosphorylated, persists. Considering the location of the phospho group in the hinge region of the ε subunit between the α and β domains, it is interesting to speculate that extension of the unstructured linker occurs in response to phosphorylation, providing a rational for the significant reduction in the intensity of this cross-link in the dephosphorylated form of the enzyme.

**Figure 7:**
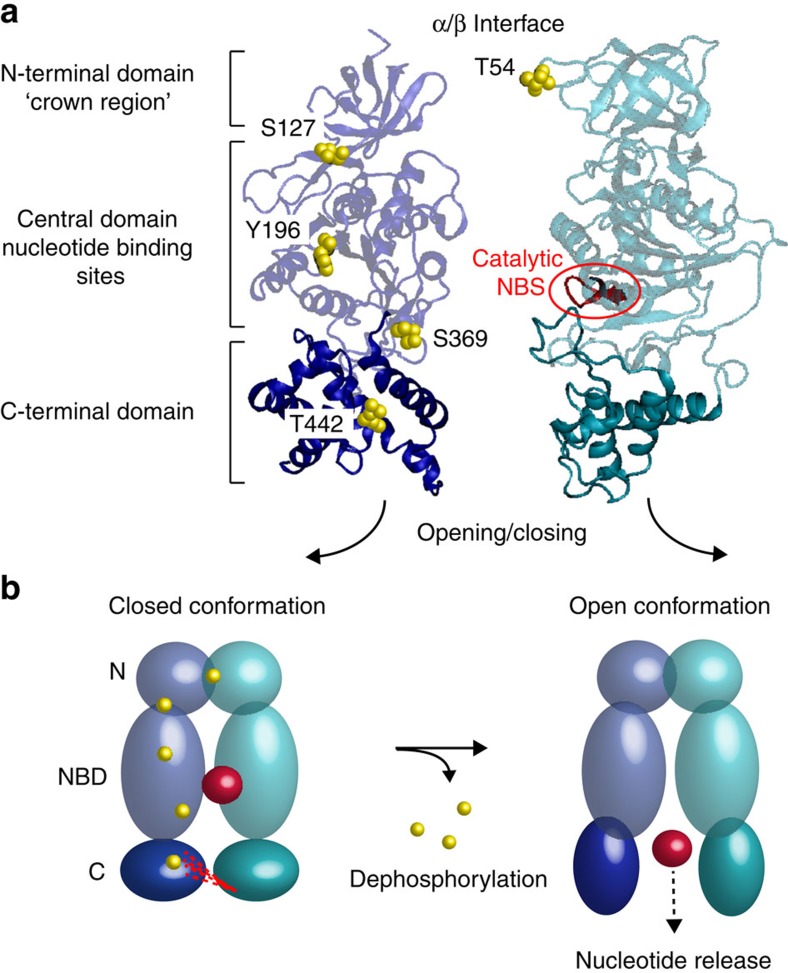
Proposed mechanism for phosphorylation-dependent nucleotide release. (**a**) The α and β subunits are shown separated with three domains labelled: the N-terminal domain (‘crown region’), the central nucleotide-binding domain and the C-terminal domain. Phosphosites are shown in yellow and are labelled with the respective residue number. The catalytic binding site of β is shown in red. (**b**) Schematic of the phosphorylation-dependent conformational changes. The three domains of the α (blue) and β (green) subunits, the phosphorylation sites (yellow) and nucleotide (red) are shown. Cross-links that are decreased following dephosphorylation are shown (dashed lines). Conformational changes in the C-terminal domains of α and β occur upon dephosphorylation, leading to nucleotide release. NBS, nucleotide binding sites.

Of significance for nucleotide occupancy are changes in cross-links at the base of subunits α and β (Fig. 6). Six cross-links occur between the unstructured loops of the β domains and the C-terminal domain of the α subunits, four are decreased five- to tenfold and two cross-links show greater than tenfold reduction upon dephosphorylation (Fig. 6 and Supplementary Table S9). This is consistent with a concerted movement of either or both of the C-terminal domains of the α and β subunits (Fig. 7a). Overall, given the location of the phosphosites at the interface, the significant reduction in cross-links at the bases of the subunits and the reduction in nucleotide occupancy upon dephosphorylation, these data strongly suggest a role for phosphorylation in regulating interactions within the α/β interface, which in turn affect nucleotide release or exchange (Fig. 7b).

## Discussion

We have transmitted intact cATPases through a mass spectrometer, preserving subunit interactions between the membrane base and the soluble head. Manipulating solution conditions enabled us to define stable subcomplexes and to delineate interactions between the head and the base. Dissolution of the lipid plug revealed the presence of the most abundant sulpholipid specific for plant membranes, and found tightly bound to cATPase previously^26^. Although only a minor component of thylakoid membranes (8%), it accounts for 90% of the lipid content in cATPase. Attempts to exchange lipids with other phospholipids or to remove them from cATPase failed, implying that the lipids are an integral part of the membrane ring. The identity of the lipid, together with the stoichiometry of binding, enabled us to model the plug within the membrane ring. These results add to the growing evidence that many rotary ATPases adapt the lipid environment to produce tight interactions within the central rotor. In the case of the cATPase, the lipid plug reduces the orifice to ≤10 Å diameter, considerably smaller than for the corresponding lipid-lined rings of the V-type ATPases (≈40 Å)^27^. The two terminal helices of the γ subunit (≈20 Å), accommodated within the lipid-lined ring of the cATPase, imply that annular lipids form an adaptable closure around the γ subunit, aiding rotation and sealing the membrane.

Subunit interfaces were investigated using chemical cross-linking, revealing two conformations of ε, one with the C-terminal domain proximal to the membrane ring and the other close to β. Cross-links between stator subunits I and II allowed their relative positions to be delineated with respect to each other, as well as to α and the C-terminal β loop. Comparing our model with the X-ray structure of the corresponding complex of the bovine and *E. coli* ATPases^28^, we find that the two stators and the oligomycin sensitivity-conferring protein (subunit δ in the cATPase) contact α directly, but not the structured regions of β analogous to those defined for cATPase, implying that the interactions of the stators in bovine, *E. coli* and cATPase are similar.

Multiple PTMs (oxidation, acetylation and phosphorylation) were defined for the first time in cATPase from *Spinacia oleracea.* We focused on the nine phosphorylation sites, a subset of which were identified in *Arabidopsis thaliana*^29^, suggesting their conservation. Dephosphorylation showed a reduction in the overall stability of the complex with facile losses of subunits I, II, III, IV, ε and δ. Eight phosphosites were located, either within known structural regions or homology models of protein subunits. One was located in the hinge region of ε and two at either end of subunit II, encompassing the span of its interactions with the head. Intriguingly, five phospho groups align along one face of the α/β interface. Comparing cross-links following dephosphorylation, we find significant reductions in cross-links along the α/β interface, between ε and α/β, and the stator subunits I/II. Importantly, we also show significant reduction in cross-links at the C terminus of the α/β-subunit interface, mediated by the long unstructured loop of the C terminus of the β subunit.

The phosphorylation site in ε at the hinge region is likely important for initiating extension of the C-terminal helix to the F_1_ head. This movement was identified in two independent experiments: the first as a gas-phase dissociation product in which ε was observed attached directly to the head in the absence of γ; the second via cross-linking experiments in which the C terminus cross-linked to the head and to the base. These two populations imply that the cATPase examined here contains both active and inactive forms. From a regulatory viewpoint, changes in cross-linking of ε are consistent with movement to prevent rotation of the soluble head when ATP levels are depleted as proposed previously for other ATPases^12^,^30^,^31^,^32^,^33^. In previous MS experiments to induce this conformational switch, solutions were deprived of ATP^12^. Moreover, as dephosphorylation reduces cross-links to the head, whereas interactions with the membrane ring remain constant, it is likely that phosphorylation is linked with inactivation of the cATPase via prevention of rotation.

Considering the role of phosphosites along the α/β interface, dephosphorylation leads to a reduction in cross-links, enhancing conformational fluctuations that allow depletion of nucleotides and facilitating access to their binding sites. Given that ATP synthesis is rapid (≈300 ATP s^−1^)^34^ compared with slower rates suggested for phosphorylation/dephosphorylation (≈5 s^−1^)^35^, phosphosites are likely important for stability rather than regulation. For longer time spans, such as in the hours of darkness, or activation following exposure to light, regulation via phosphorylation/dephosphorylation cannot be excluded as observed for other photosynthetic complexes^36^. The kinase CKII is proposed to phosphorylate the β subunit of the cATPase^37^, preferentially in the dark^38^ when ATP synthesis rates are reduced. Although this phosphosite was not observed in our study, as cATPase was grown and collected in the presence of light, it implies that phosphorylation has an inhibitory role, preventing unnecessary production of ATP. This is in accord with the X-ray structure of the F_1_ head in which residues from Glu383 to Phe430 were predicted to be involved in conformational changes associated with nucleotide binding and catalytic turnover^9^. The changes in cross-linking observed here provide the first direct evidence for the role of phosphorylation in preventing dynamic fluctuations that facilitate nucleotide exchange. Dephosphorylation conversely reduces cross-linking at the C terminus of the α/β interface, allowing access to the nucleotide binding sites for nucleotide exchange.

Understanding the location of phosphorylation sites is not only important with respect to function but also in terms of their likely fluctuation *in vivo.* In a recent survey of the phosphoproteome, it was shown that phosphosites within loops are more likely to fluctuate during the cell cycle than those in structured regions^39^. The single phosphosite in the ε subunit is in the unstructured hinge region, between the N- and C-terminal domains, implying that its phosphorylation status fluctuates in line with its proposed regulatory mechanism. By contrast, the five phosphosites located equidistant along one face of the α/β interface are in structured regions and are therefore expected to fluctuate less. A mechanism in which dephosphorylation has a synergistic effect on neighbouring phosphosites could control this critical interface. In such a mechanism, C-terminal regions of the α/β interface accessible to phosphatases facilitate opening of the interface. This would have a synergistic effect on neighbouring sites, promoting conformational changes that allow access to nucleotide binding sites. Taken together, therefore, phosphorylation is likely to have critical roles in regulating ATP synthesis, not only through prevention of rotation of the F_1_ head, through interactions with ε prompted by phosphorylation, but also through controlling access to nucleotide-binding sites.

More generally, this novel comparative cross-linking strategy has enabled us to define the effects of phosphorylation on dynamic subunit interactions that occur within a membrane motor. By combining this strategy with the study of intact complexes, in which nucleotide and lipid binding can be assessed, we provide a comprehensive view of the composition, dynamics and regulation of this cellular machine. The prospects for this combination to unravel regulation by phosphorylation, and associated conformational changes is likely to have widespread significance for structural biology. Particularly important is the potential for unravelling the synergy that exists between multiple phosphorylation sites and their effects on functional regulation.

## Methods

### Purification of F_1_F_O_ chloroplast ATP synthase

F_1_F_O_ chloroplast ATP synthase was extracted from spinach leaves as described previously^40^. See Supplementary Methods for detailed protocol.

### Protein identification

Proteins were digested with Trypsin in-gel as described^41^. Tryptic peptides were separated by reversed-phase nanoLC (DionexUltiMate 3000 RSLC nano System, Thermo Scientific) and directly analysed in a LTQ-Orbitrap XL hybrid mass spectrometer (Thermo Scientific). Proteins were identified by database search against NCBInr using the Mascot search engine (see Supplementary Methods for experimental details).

### Lipid analysis

cATPase proteins were digested with Trypsin and the peptide/lipid mixture was separated by reversed-phase nanoLC, and directly analysed in a LTQ-Orbitrap XL hybrid mass spectrometer. Lipids were identified manually from MSMS spectra (see Supplementary Methods for experimental details).

### Docking of lipids into the membrane ring

A three-dimensional model structure for sulphoquinovosyl diacylglycerol was generated by performing a molecular dynamics simulation using a molecular mechanics force field^42^ as implemented in ChemDraw12. The simulation was run for 10,000 steps over a linear temperature gradient of 300−500 K. The output structure for the lipid and a monomer of the cATPase membrane ring (pdb ID 2W5J) were subsequently subjected for docking analysis using an in-house approach for generation of protein complexes from their individual components^43^. First, the conformational space for the monomer–lipid complex was sampled using a Monte Carlo search followed by a conjugated gradient optimization step using the Integrative Modelling Platform^44^. Overall, 10,000 models were generated. Next, we screened all structures for satisfaction of a distance restraint (<5 Å) defined by the negatively charged sulpholipid and the positively charged arginine of subunit III at the top of the membrane ring on the inside face. Four, closely related structures (root mean squared deviation difference <3 Å) were found to satisfy this criterion. By selecting the structure with the smallest distance between the lipid and arginine (4.03 Å), and after refinement by an energy minimization step, we propose a model for the sulpholipid docked onto the monomer from the ring. The 14 sulpholipids bound to the ring were generated using a transformation matrix derived from the atomic coordinates of individual subunits in the chloroplast ring (pdb ID 2W5J).

### Determination of accurate protein masses by denaturing LC–MS

Accurate masses of protein subunits were determined by denaturing LC–MS. The proteins were separated by nano-flow liquid chromatography (DionexUltiMate 3000 RSLC nano System, Thermo Scientific; mobile phase A: 0.05% (v/v) trifluoroacetic acid (TFA) mobile phase B: 50% (v/v) acetonitrile/50% (v/v) isopropanol/0.04% (v/v) TFA) and directly eluted into a QSTAR XL mass spectrometer (ABSciex). The protein sample was spiked with TFA to a final concentration of 0.05% (v/v) and loaded onto a monolithic PS-DVB column (100 μm × 25 cm, Thermo Scientific). Protein subunits were separated with a flow of 600 nl min^−1^ by applying a linear gradient from 8 to 98% solvent B over 30 min, and delivered to a QSTAR XL mass spectrometer. Typical mass spectrometric conditions were as follows: ion spray voltage 2,980 V, declustering potential 75 V and collision energy 17 V.

### MS of intact cATPase complexes

Aliquots of cATPase in *n*-dodecyl-β-D-maltopyranoside-containing buffer were exchanged against 200 mM ammonium acetate using Micro Bio-spin 6 columns (Bio Rad). Spectra were acquired on a Q-ToF II mass spectrometer (Waters) modified for high masses^20^ using in-house prepared gold-coated glass capillaries^45^. Optimized instrument parameters were as follows: capillary voltage 1.7 kV, cone voltage 190 V, extractor 5 V, source backing pressure 7–10 mbar and a collision cell pressure of 10 psi. Collision cell energy was 100–200 V. All spectra were processed and complexes were assigned using in-house software Massign^22^.

### Protein–protein cross-linking

Ten microlitre of a 1:1 mixture of 2.5 mM deuterated (d4) and 2.5 mM non-deuterated (d0) BS3 were added to 20 μl aliquot of cATPase. The optimized ratio of cross-linker to protein was found by investigating of a series of protein complexes. The reaction mixture was incubated for 1 h at room temperature and 350 r.p.m. in a thermomixer. The cross-linked sample and the control were subsequently loaded onto a NuPAGE gel. Protein bands were cut, digested in gel and analysed by LC–MS/MS as described (see above). Potential cross-links were identified by using the MassMatrix Database Search Engine^46^,^47^,^48^,^49^. Search parameters were as follows: peptides were defined to be tryptic with a maximum of two missed cleavage sites. Carbamidomethylation of cysteines and oxidation of methionine residues were allowed as variable modifications. The mass accuracy filter was 10 p.p.m. for precursor ions and 0.8 Da for fragment ions. Minimum pp and pp2 values were 5.0 and minimum pp_tag_ was 1.3. Maximum number of cross-links per peptide was one. All searches were performed against deuterated and non-deuterated cross-linked proteins. Potential cross-links were validated manually by (i) checking the presence of corresponding peak pairs in the MS spectra generated by the d4/d0-BS3 mixture and (ii) by the quality of the MSMS spectrum.

For comparative cross-linking, equal amounts of untreated and dephosphorylated cATPase were cross-linked separately with 2.5 mM non-deuterated and deuterated BS3, and were subsequently pooled. After validation of the potential cross-linked peptides, extracted ion chromatograms for the light and heavy cross-links, respectively, were generated. The two different states of the cATPase were compared using the area of the extracted ion chromatograms (see Supplementary Fig. S18).

### Homology modelling

Homology models of γ, ε and δ were generated using the MODELLER web server ( https://modbase.compbio.ucsf.edu/scgi/modweb.cgi). Models were selected from the list of calculated models according to their ModPipe Protein Quality Score, which is a measure for reliability of the models. Homology models of peripheral stalk subunits I and II were generated using the 3D-JIGSAW web server ( http://bmm.cancerresearchuk.org/~populus/). The models were selected according to their similarity to available crystal structures of the peripheral stalk proteins from other species.

### Analysis of PTMs

Proteins were digested in gel^41^, and peptides generated were separated by reversed-phase nanoLC and analysed directly in a LTQ-Orbitrap mass spectrometer. Phosphopeptides were enriched using TiO_2_ and analysed by collision induced dissociation with multistage activation. Analysis of acetylation, methylation and trimethylation of peptides was performed by higher energy collision dissociation fragmentation. Proteins and their PTMs were identified by database search against NCBInr database using Mascot search engine (see Supplementary Methods for experimental details).

### Dephosphorylation of the cATPase

Intact cATPase complexes were dephosphorylated using calf intestinal alkaline phosphatase (New England Biolabs). Two to four microlitre calf intestinal alkaline phosphatase were added to a volume of 20 μl cATPase. Incubation was performed at 37 °C and stopped on ice. The buffer was subsequently exchanged to 200 mM ammonium acetate using Micro Bio-spin 6 columns (Bio Rad).

## Author contributions

C.S. designed and conducted all experiments, analysed the data and wrote the paper; M.Z. and H.M. performed some MS experiments; N.M. analysed the data; A.P. performed lipid modelling; C.V.R. wrote the paper and directed the research.

## Additional information

**How to cite this article:** Schmidt, C. *et al.* Comparative cross-linking and mass spectrometry of an intact F-type ATPase suggest a role for phosphorylation. *Nat. Commun.* 4:1985 doi: 10.1038/ncomms2985 (2013).

## Supplementary Material

Supplementary Information Supplementary Figures S1-S17, Supplementary Tables S1-S9, Supplementary Methods and Supplementary References
