# Supplementary material for: Comparative cross-linking and mass spectrometry of an intact F-type ATPase suggest a role for phosphorylation
Source: Nat Commun. 2013 Jun 12;4:1985. doi: 10.1038/ncomms2985 (PMC3709506; doi:10.1038/ncomms2985)
Supplement: Supplementary Information — Supplementary Figures S1-S17, Supplementary Tables S1-S9, Supplementary Methods and Supplementary References [file ncomms2985-s1.pdf]

Supplementary Information

Comparative crosslinking and mass spectrometry of an intact F-type ATPase suggests a role for phosphorylation

Carla Schmidt, Min Zhou, Hazel Marriott, Nina Morgner, Argyris Politis and Carol V. Robinson

Department of Chemistry, Physical and Theoretical Chemistry Laboratory, University of Oxford, Oxford OX1 3QZ, UK

* To whom correspondence should be addressed:

e-mail: carol.robinson@chem.ox.ac.uk

Supplementary Figures

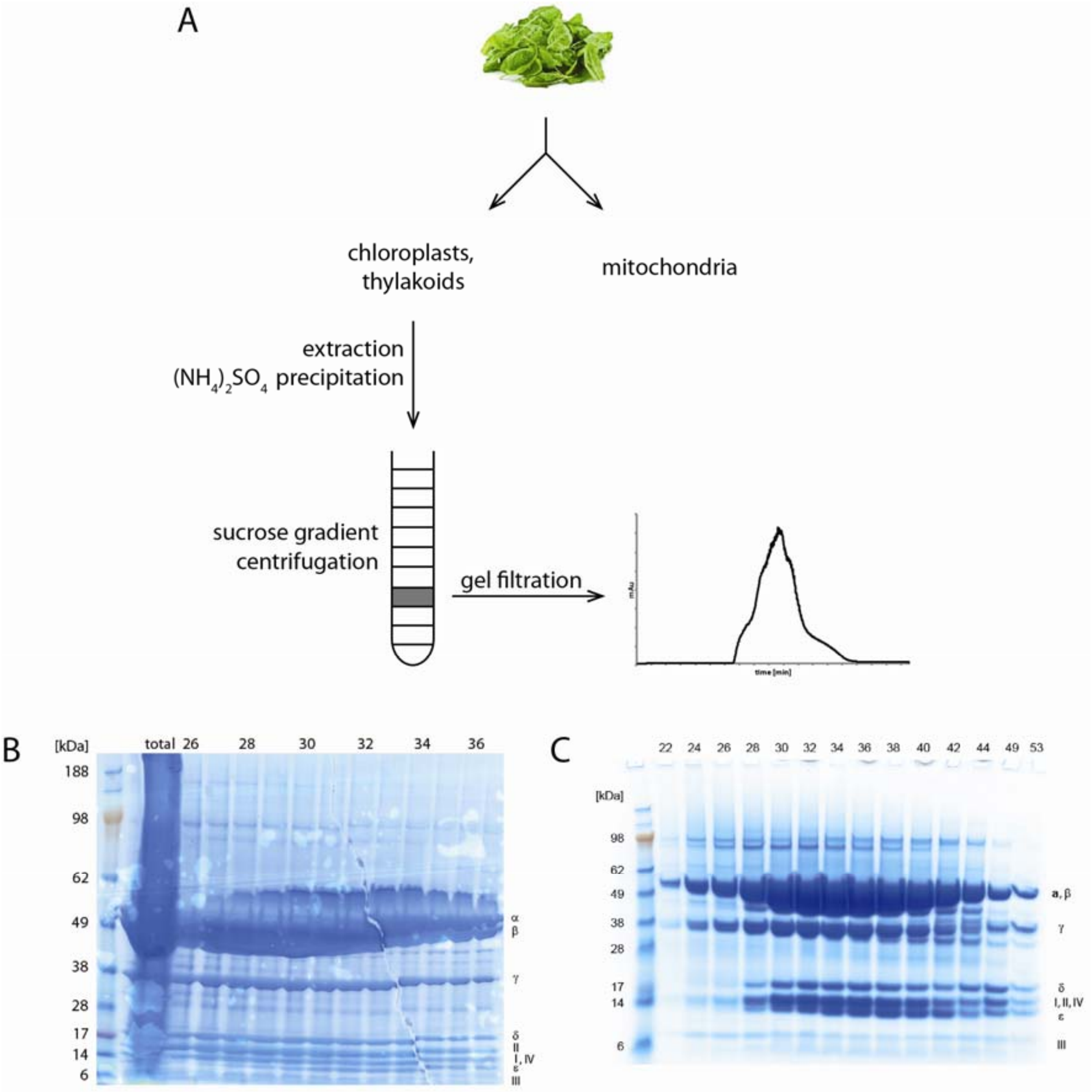

Supplementary Figure S1: Purification of the cATPase from spinach leaves. (A) The chloroplasts were isolated from spinach leaves and the cATPase and other membrane complexes were extracted from thylakoid membranes. The cATPase was then further purified by fractionated ammonium sulfate precipitation and sucrose gradient centrifugation. Intact cATPase complexes were separated by gel filtration. **(B)** A representative SDS-PAGE gel after sucrose gradient centrifugation. The cATPase is found in fractions 26 to 36. All 9 protein subunits are present. **(C)** A representative SDS-PAGE gel after gel filtration. The cATPase is found in fractions 28 to 42. All 9 protein subunits are present. In both gels the membrane subunit IV appears to be under-represented compared to the other proteins (B and C).

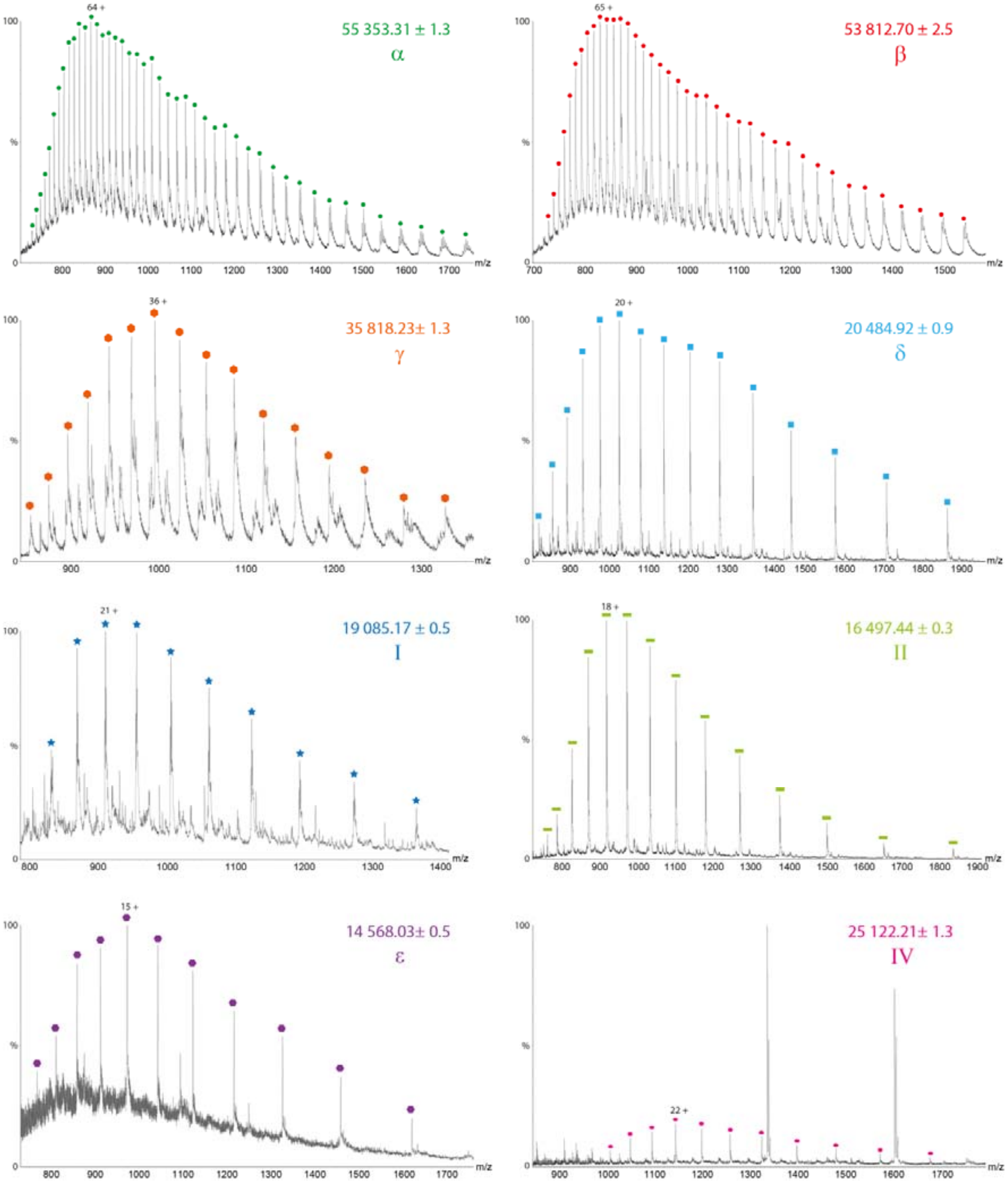

Supplementary Figure S2: Denaturing LC-MS of cATPase protein subunits for determination of protein masses. Mass spectra of α, β, γ, δ, ε, I, II and IV are shown. The accurate protein masses as determined by the charge states are given. Spectra are chosen from three replicates.

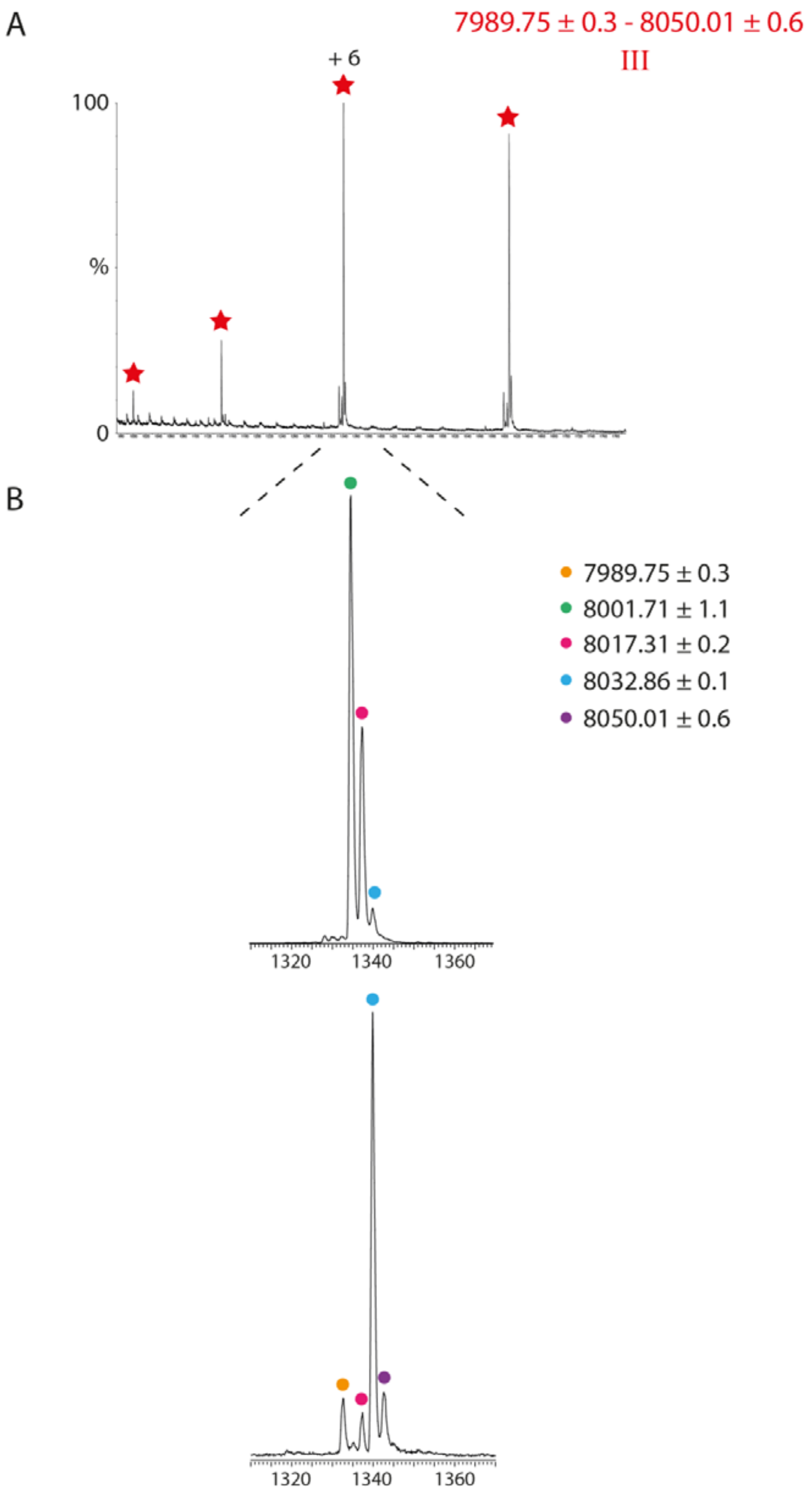

Supplementary Figure S3: Denaturing LC-MS of subunit III. (A) Subunit III shows different peak distributions. Masses between 7989.75 and 8050.01 Da have been determined. **(B)** Magnification of the most intense peak. Subunit III elutes in two peaks (upper and lower panel), both showing different peak distributions. The mass differences between the peak envelopes indicate oxidation of III. The differently modified versions are labeled with coloured circles.

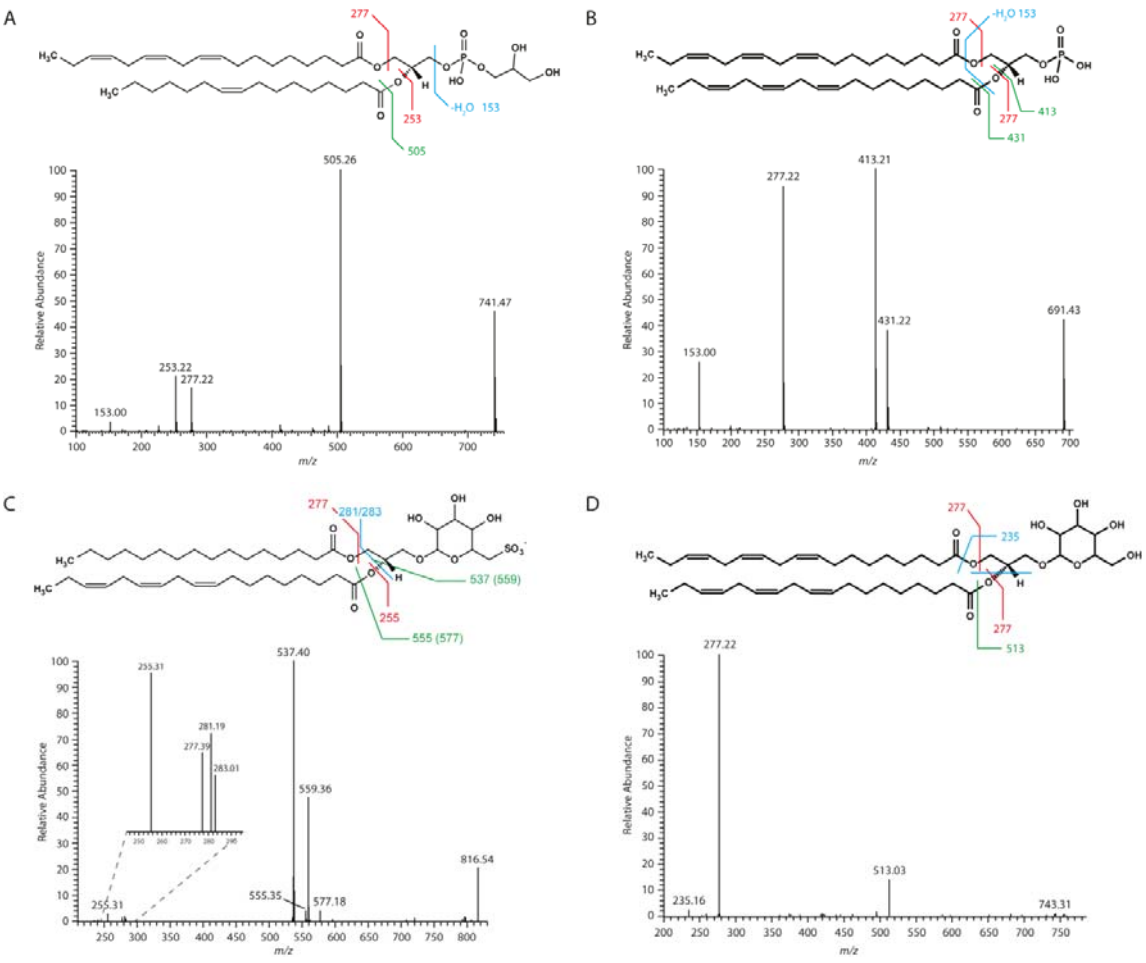

Supplementary Figure S4: Analysis of the cATPase lipid plug by LC-MSMS. Four different groups of lipids were identified. Panels A-D show mass spectra with characteristic fragmentation patterns of the most intense lipid of the respective groups. **(A)** Diacylglycerophosphoglycerol; **(B)** Diacylglycerophosphate; **(C)** Sulfoquinovosyl diacylglycerol; **(D)** Glycosyldiacylglycerol.

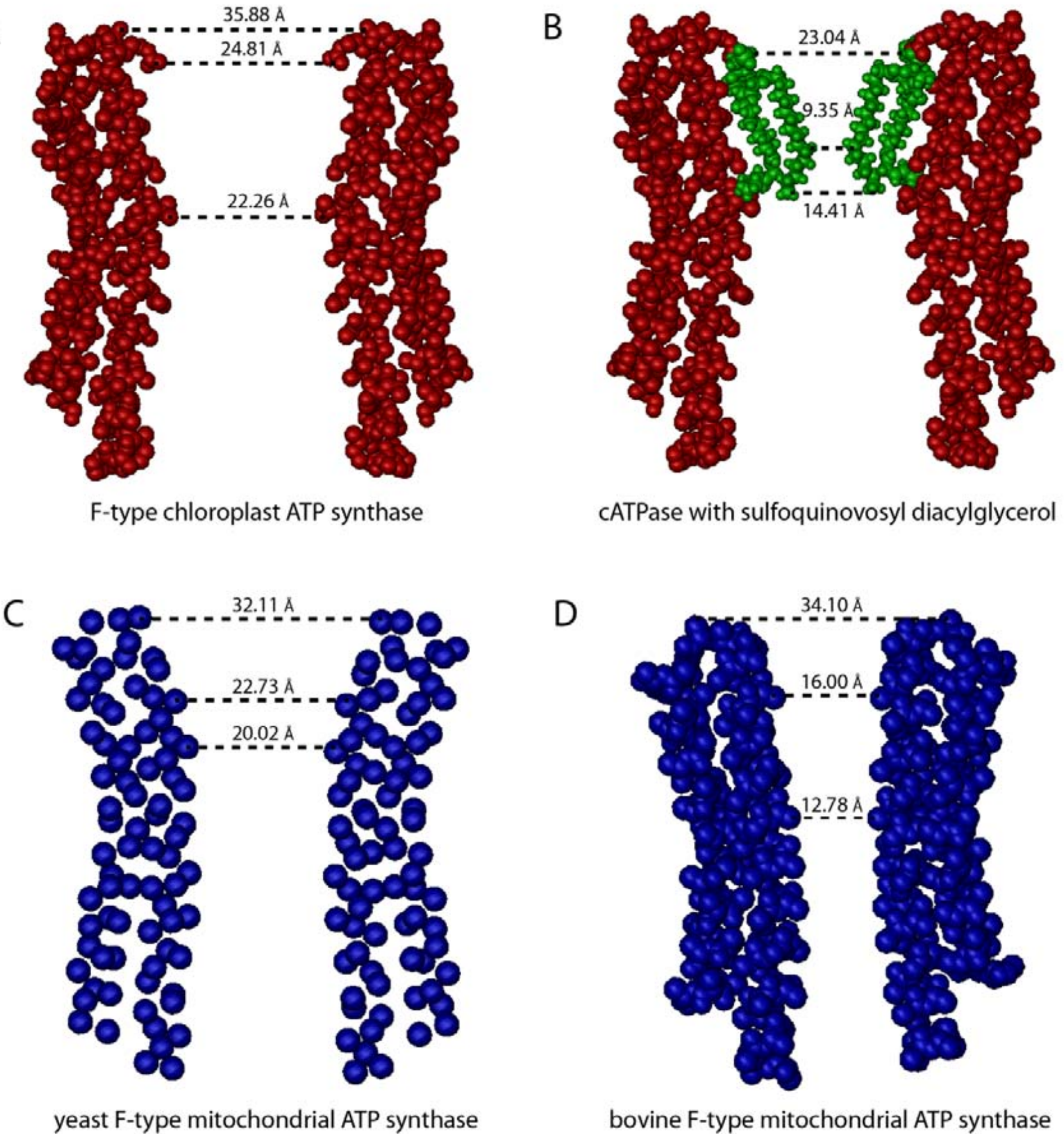

Supplementary Figure S5: Approximate dimensions of the membrane rings of F-type ATP synthases. (A) Spinach cATPase (pdb ID 2W5J). **(B)** The cATPase membrane ring with docked Sulfoquinovosyl diacylglycerol. The lipid plug reduces the central orifice of the membrane ring. **(C)** The yeast F-type mitochondrial ATPase (pdb ID 1QO1) **(D)** The bovine F-type mitochondrial ATPase (pdb ID 2XND).

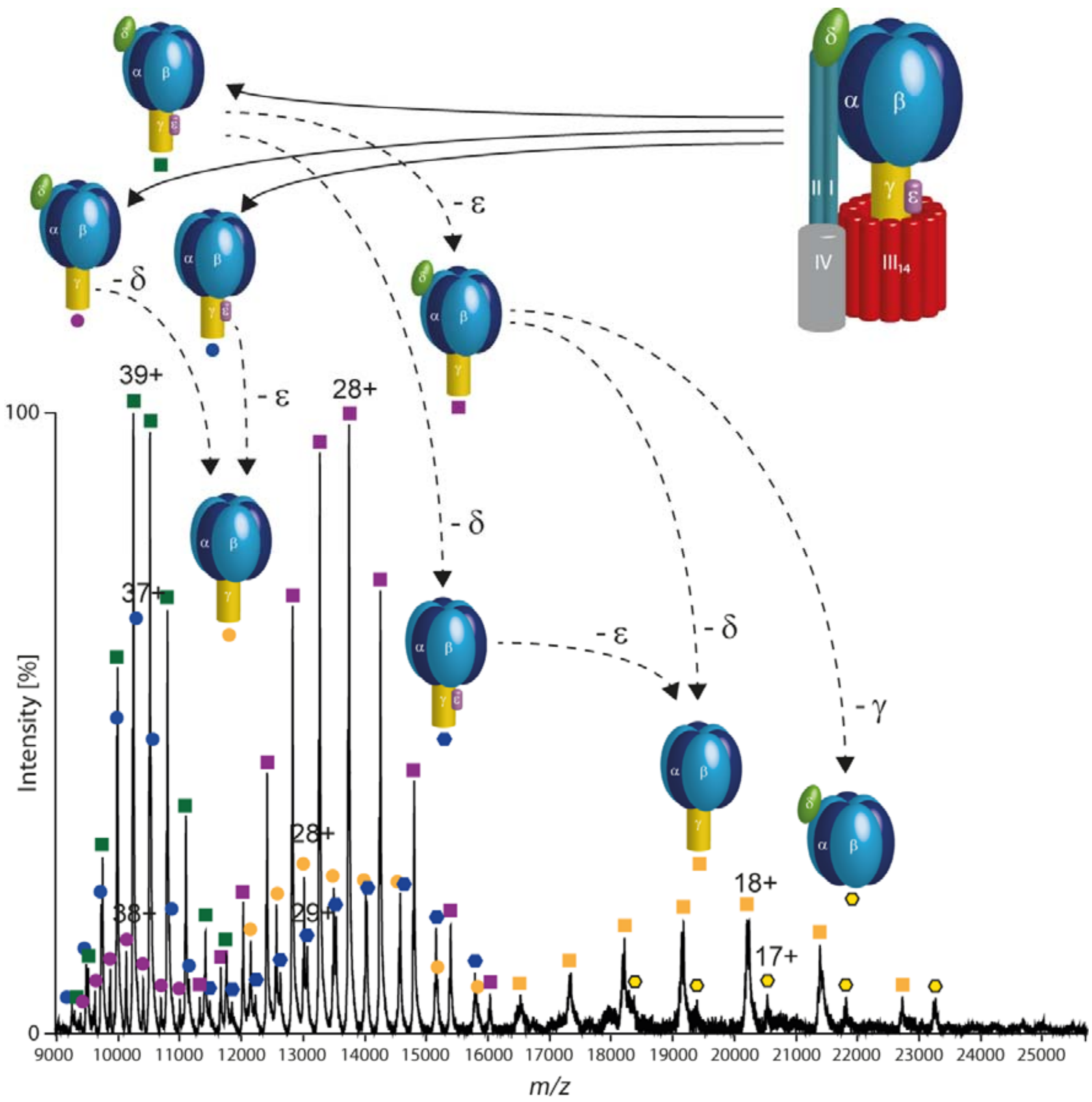

Supplementary Figure S6: Mass spectrum of the soluble F_1 cATPase ($\alpha_3\beta_3\gamma\delta\varepsilon$). Complexes stable in solution are assigned to intact F_1 (green squares), loss of δ (blue circles) and ε (purple circles) subunits. 1st and 2nd gas phase dissociation products are observed at higher *m/z*. These are assigned to the $\alpha_3\beta_3\gamma$ (orange circles and squares), $\alpha_3\beta_3\gamma\delta$ (purple squares), $\alpha_3\beta_3\gamma\varepsilon$ (blue diamonds), $\alpha_3\beta_3\delta$ subcomplexes (yellow diamonds). The spectrum represents a typical mass spectrum of the F_1 cATPase.

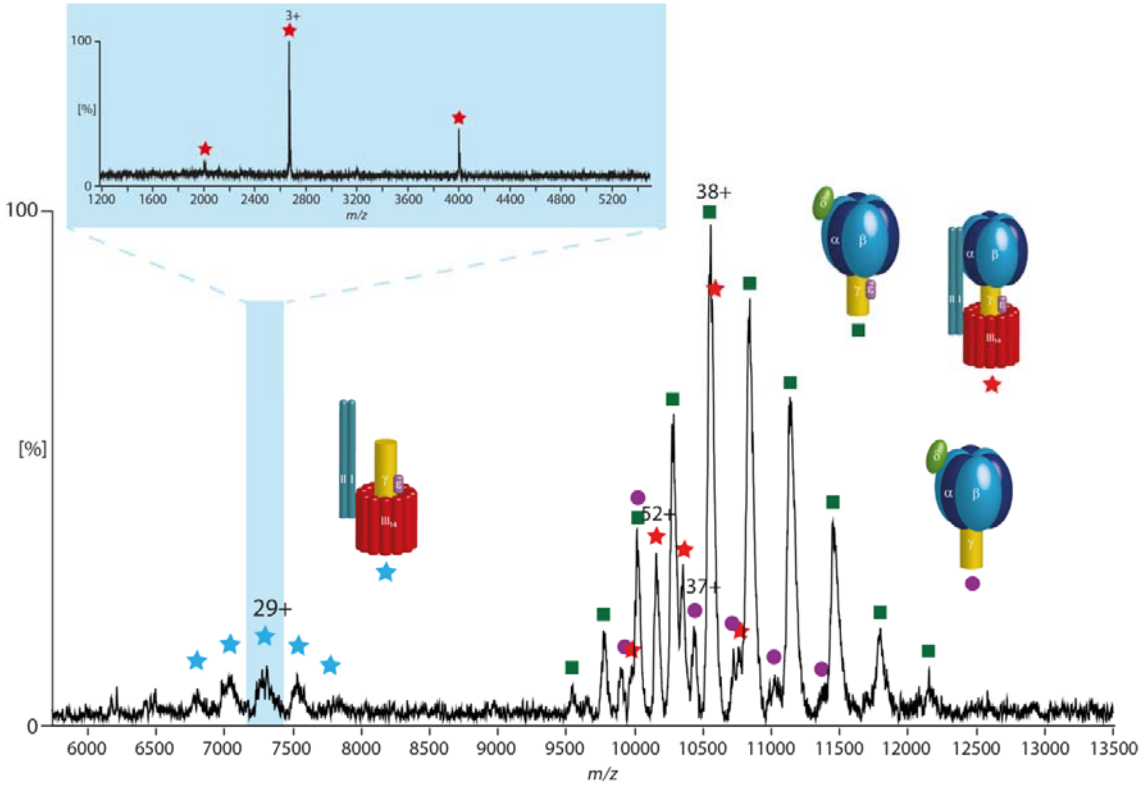

Supplementary Figure S7: A complex containing the membrane ring, the two stator subunits (I and II), γ and ε was observed. Only low intense and poorly resolved peaks could be detected for this complex presumably due to incomplete desolvation of the membrane domain. However, there is only one possible protein composition of this complex and a tandem mass spectrum confirms the presence of the membrane ring subunit III within this complex (blue insert).

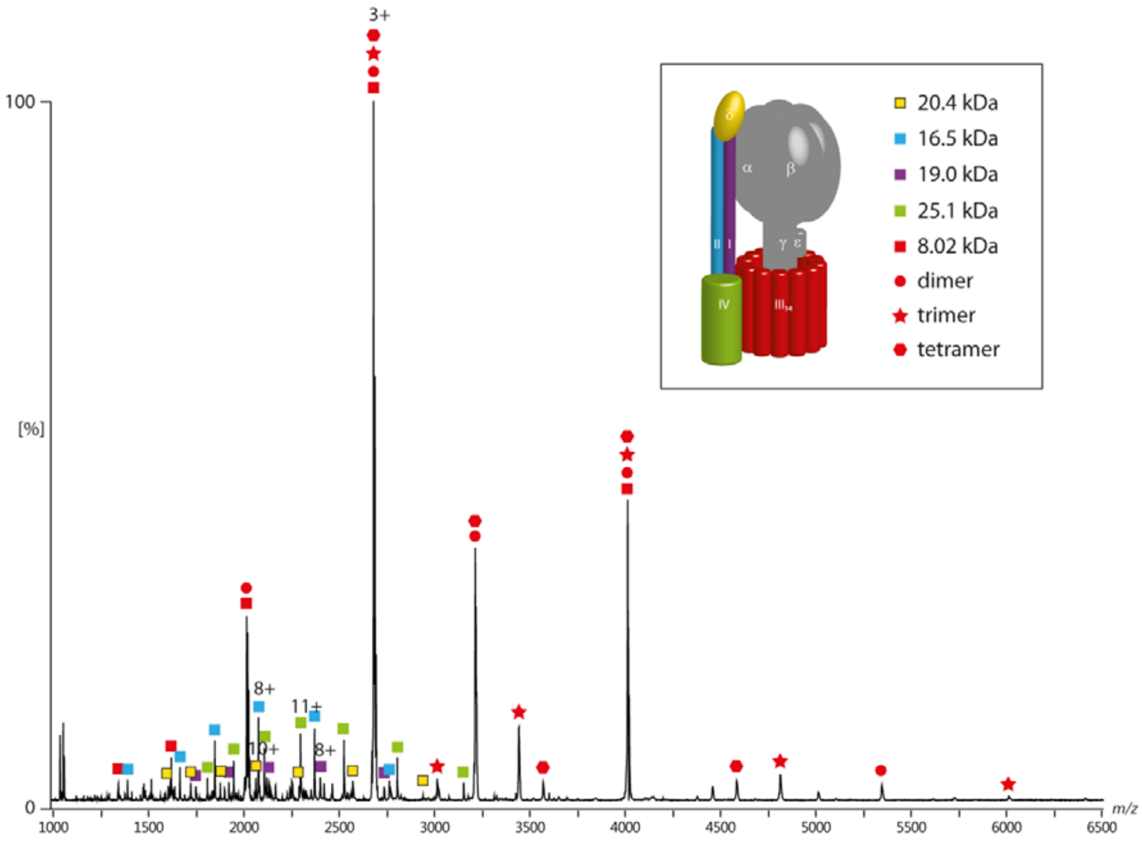

Supplementary Figure S8: Low m/z region of a typical mass spectrum of the cATPase. The low m/z region is dominated by the membrane ring subunit III (red squares) and its multimers such as dimers (red circles), trimers (red stars), and tetramers (red hexagons). Also present at lower intensities in this spectrum are subunits δ (yellow squares), I (purple squares), II (blue squares), and IV (green squares).

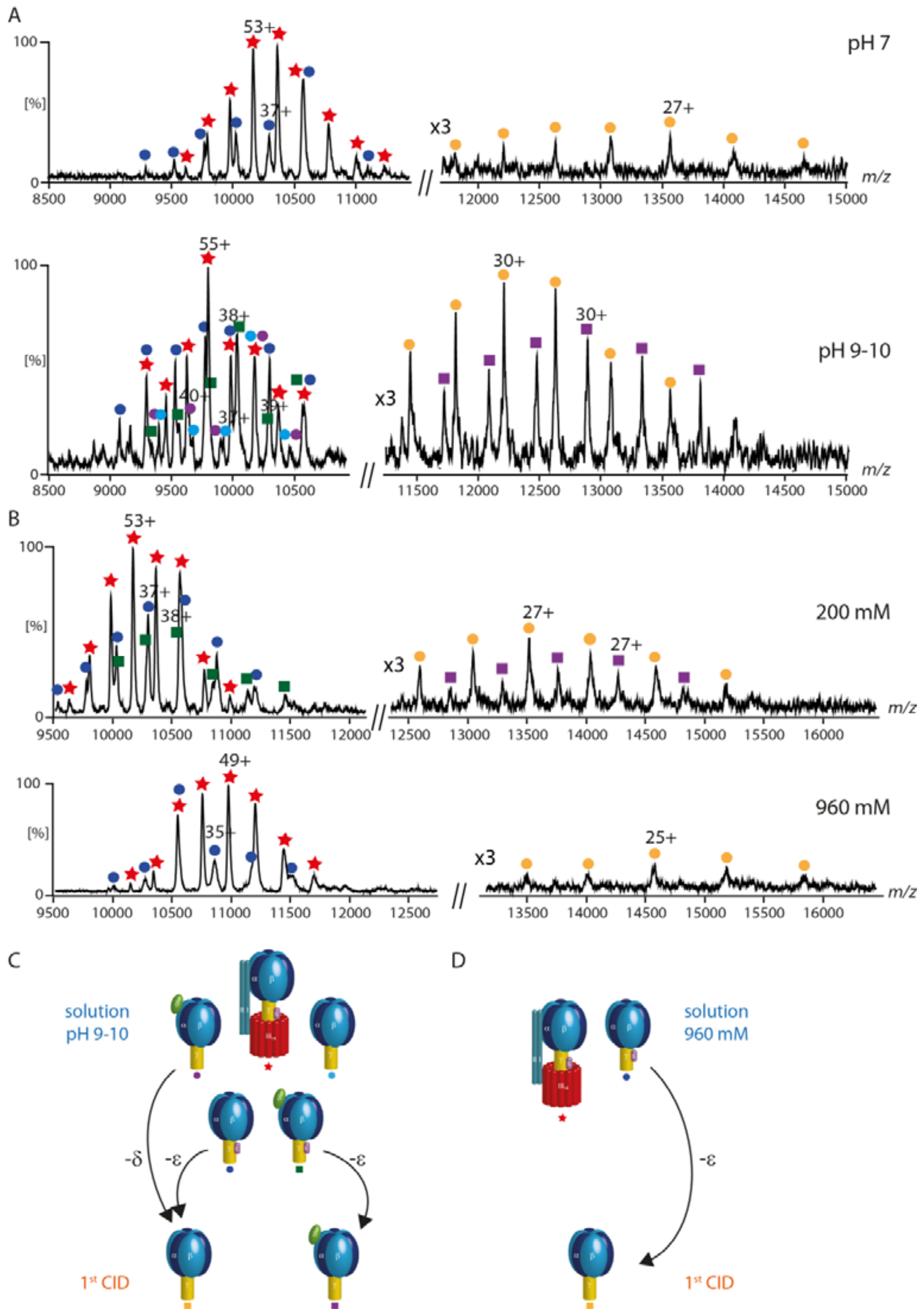

Supplementary Figure S9: In-solution disruption of cATPase complexes under basic conditions and at high ionic strength. (A) Under basic conditions (shown pH 9-10) ε and δ dissociate from the stable cATPase complexes in solution and CID products are generated more readily. **(B)** At high concentrations of ammonium acetate (shown 960 mM) the high mass complex of 537 kDa appears to be very stable while stable subcomplexes are disrupted. CID products are thus not as easy generated. **(C)** At pH 9-10 a multitude of complexes is present in solution. Most of these are generated by dissociation of ε and δ. **(D)** At 960 mM ammonium acetate the 537 kDa intact cATPase is the most stable complex. One F_1 complex is present at low intensity.

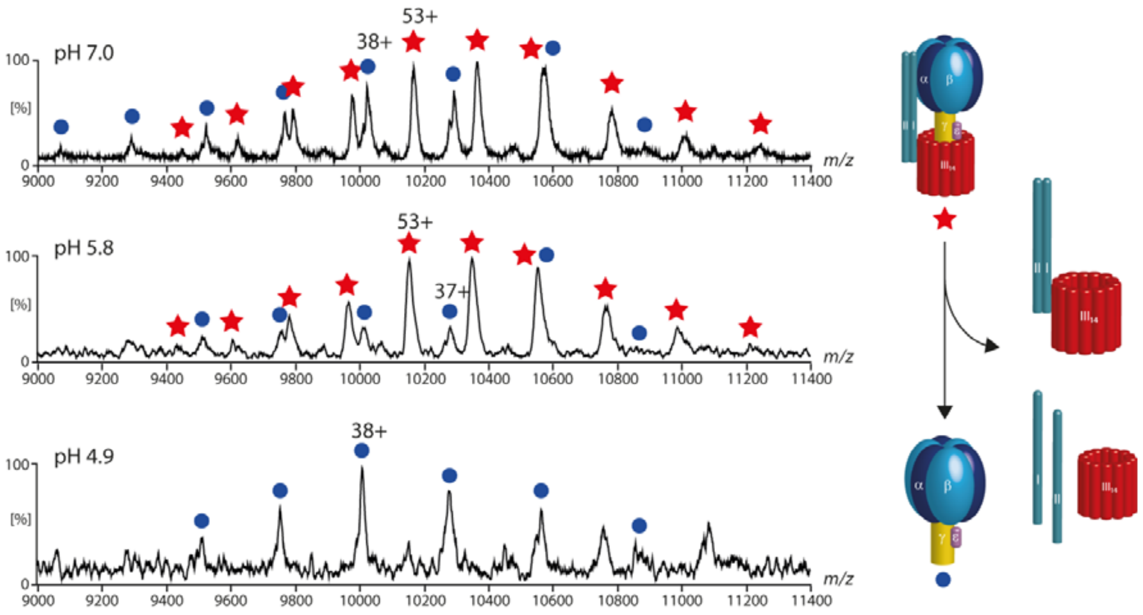

Supplementary Figure S10: In-solution disruption under acidic conditions. The intact 538 kDa complex (red stars) appears to be stable even under acidic conditions. Further decrease of the pH causes dissociation of the stalk subunits (I and II) and the membrane ring. The remaining complex at pH 4.9 is the corresponding F_1 complex.

.

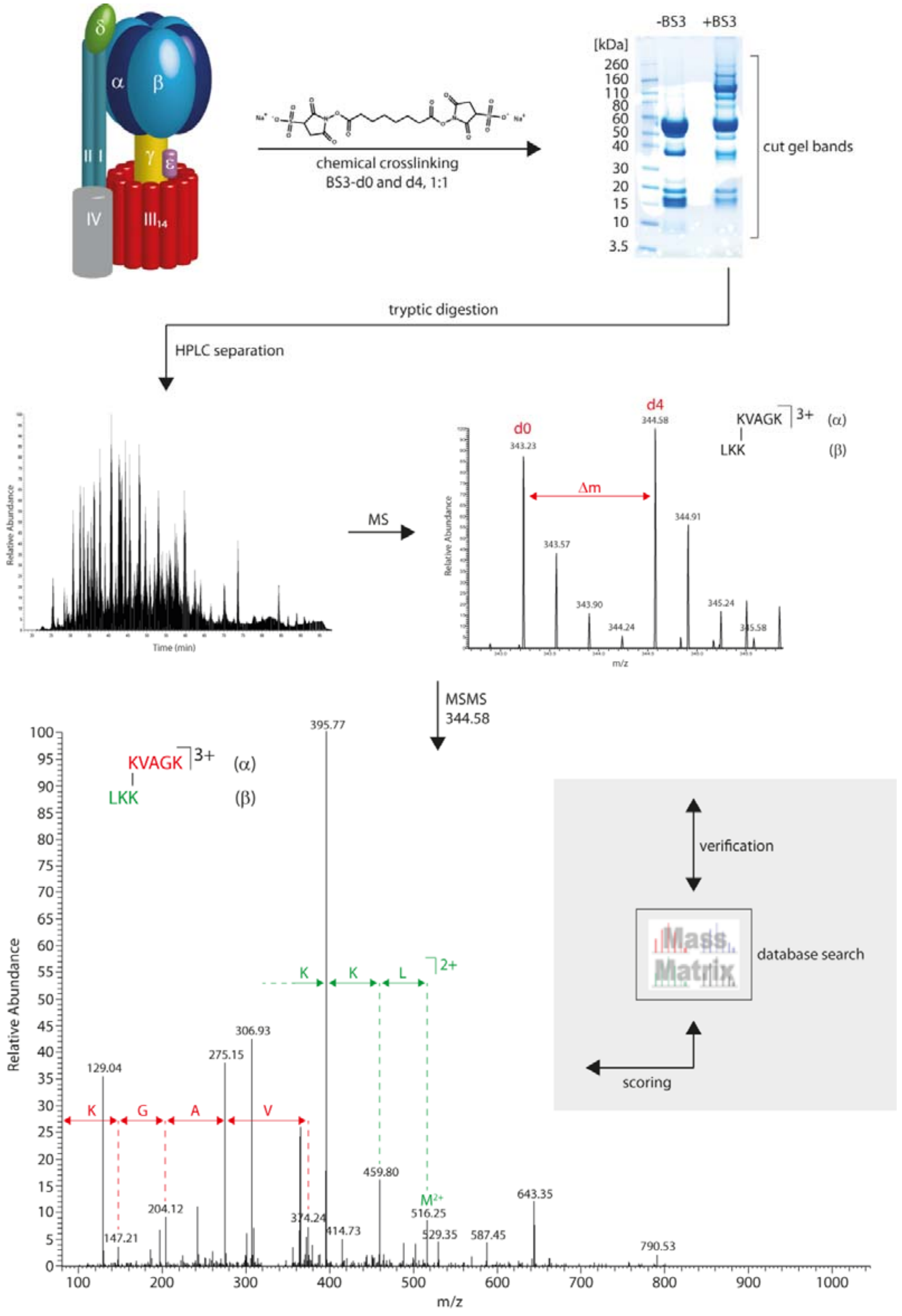

Supplementary Figure S11: Crosslinking workflow for investigation of protein-protein interactions within the cATPase. The complex(es) have been crosslinked using deuterated and non-deuterated BS3 in a 1:1 mixture. Bands were cut from the gel and proteins therein were digested. Generated peptides were analysed by nanoLC-MS/MS. Potential crosslinked peptides were identified using the MassMatrix database search engine and crosslinks were verified by their specific pattern in MS spectra (d0 and d4) and the quality of MSMS spectra (sequence information).

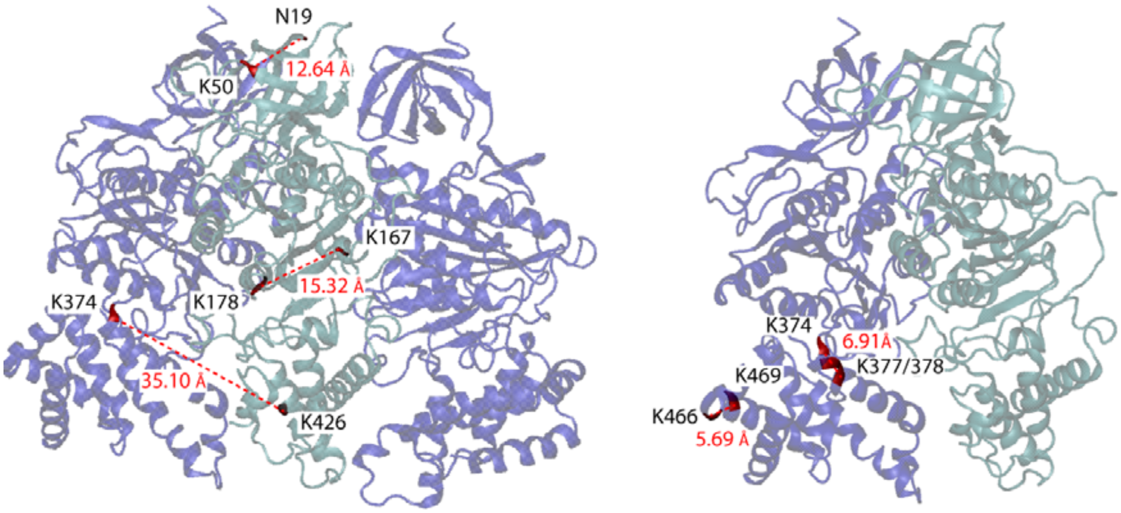

Supplementary Figure S12: Validation of identified crosslinks within the $\alpha_3\beta_3$ „head“. The crystal structure of the α and β subunits (pdb ID 1FX0) are shown in blue and cyan, respectively. Crosslinked residues are shown in red. The distances between Cα atoms of crosslinked residues are given in Å. Several crosslinks could not be shown as the crystal structure does not include the entire amino acid sequences. The crosslink between βK50 and βK18 has been replaced by βK50–N19, which is the first available residue in the crystal structure. All crosslinked residues are in reasonable distance to each other.

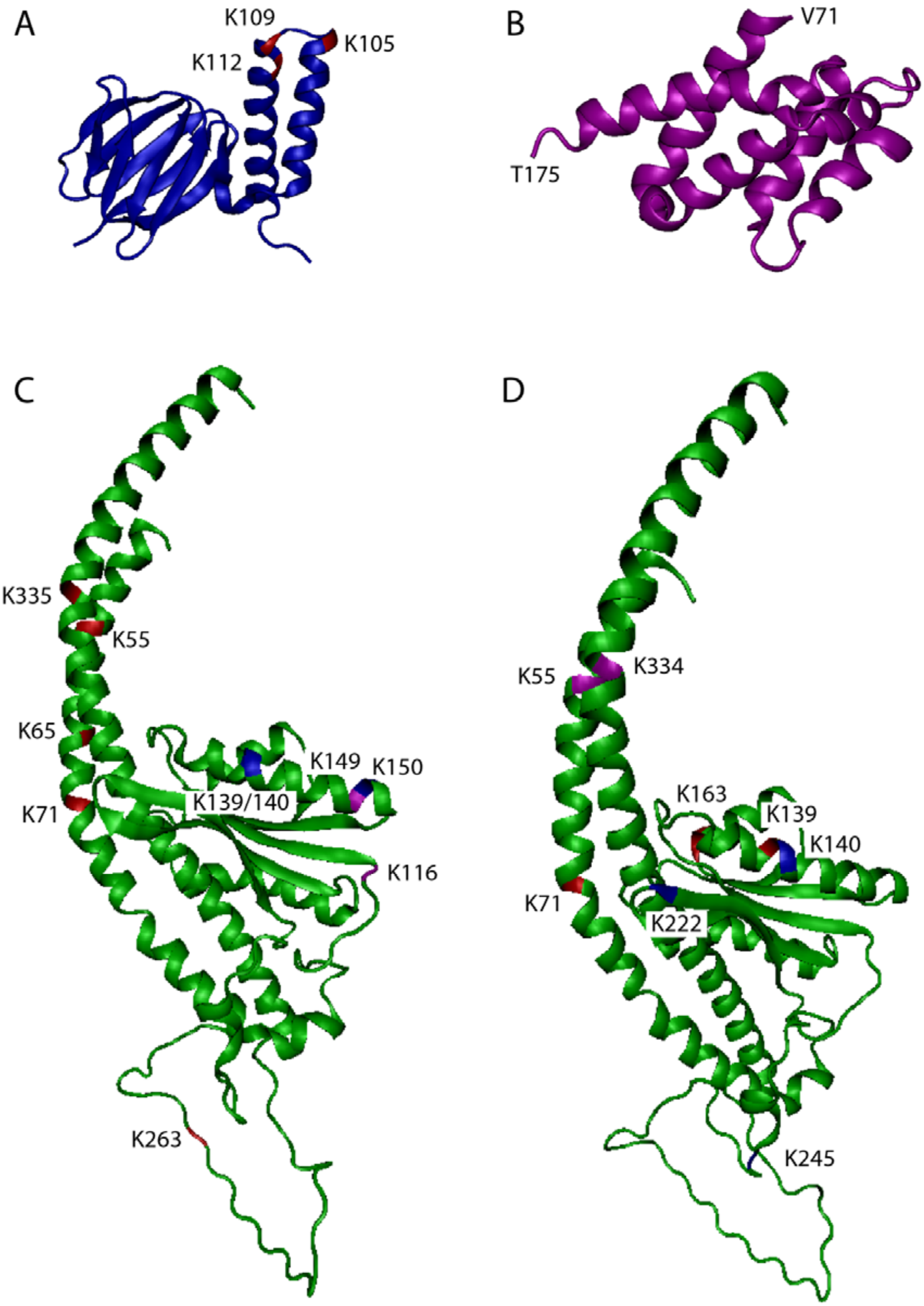

Supplementary Figure S13: Homology models of ε, δ, and γ. (A) Homology model of ε. Three lysine residues have been found to be crosslinked (red). **(B)** Homology model of δ. Only part of the protein (V71 to T175) could be modeled. The identified crosslinks are not present in the homology model (Supplementary Table 5). **(C, D)** Different views of the homology model of γ. Intra-protein crosslinks are shown in red, magenta and blue. For clarity, intra-peptide crosslinks are not shown.

A

```
Conf: 958999999995188877779788999999999999999999999999999999999999
Pred: CHHHHHHHHHHHCCCCCCCCCCHHHHHHHHHHHHHHHHHHHHHHHHHHHHHHHHHHHHHH
  AA: MKNVTDSFVFLGHWPSAGSFGFNTDILATNLINLSVVLGVLIFFGKGVLSDLLDNRKQRI
              10        20        30        40        50        60

Conf: 999999999999999999999999999999999999999999999999999999999999
Pred: HHHHHHHHHHHHHHHHHHHHHHHHHHHHHHHHHHHHHHHHHHHHHHHHHHHHHHHHHHHH
  AA: LNTIRNSEELRGKAIEQLEKARARLKKVEMDADQFRVNGYSEIEREKMNLINSTYKTLEQ
              70        80        90       100       110       120

Conf: 999999999999999999999999999999999999973899999999999999828974
Pred: HHHHHHHHHHHHHHHHHHHHHHHHHHHHHHHHHHHHHHCCHHHHHHHHHHHHHHHHCCHH
  AA: FENYKNETIQFEQQKAINQVRQRVFQQALQGALGTLNSCLNNELHLRTINANIGMFGAMN
             130       140       150       160       170       180

Conf: 1479
Pred: HCCC
  AA: EITD
```

B

```
Conf: 931232018987568999999999998876768733579101862222249999999999
Pred: CCHHHEECCCCCCCCCCCCCCCCCCCCCCCCCCCCCCCCCCCHHHCCHHHHHHHHHHHHH
  AA: MANMLVASSSKTLPTTTTTTITPKPKFPLLKTPLLKLSPPQLPPLKHLNLSVLKSAAITA
              10        20        30        40        50        60

Conf: 999999999998889984129901019899999999999999999999999999999999
Pred: HHHHHHHHHHHHHHHHHHHCCCEECCCHHHHHHHHHHHHHHHHHHHHHHHHHHHHHHHHH
  AA: TPLTLSFLLPYPSLAEEIEKASLFDFNLTLPIIMAEFLFLMFALDKIYYTPLGDFMDKRD
              70        80        90       100       110       120

Conf: 999999999999999999999999999999999999999999999999999999999999
Pred: HHHHHHHHHHHHHHHHHHHHHHHHHHHHHHHHHHHHHHHHHHHHHHHHHHHHHHHHHHHH
  AA: ASIKEQLSGVKDTSSEVKQLEEQANAVMRAARAEISAALNKMKKETQLEVEAKLAEGRKK
             130       140       150       160       170       180

Conf: 999999999999999999999999999999999999998519
Pred: HHHHHHHHHHHHHHHHHHHHHHHHHHHHHHHHHHHHHHHHCC
  AA: IEVELQEALGSLEQQKEDTIKSLDSQISALSDDIVKKVLPVS
             190       200       210       220
```

Supplementary Figure S14: Secondary structure prediction of the two stalk subunits I (A) and II (B) as obtained from 3D-JIGSAW webserver (PSIPRED v2.5). Conf: Confidence (0 = low, 9 = high), Pred: Predicted secondary structure (H = helix, C = coil), AA: amino acid sequence. The sequences of subunits I (A) and II (B) and predicted secondary structures are shown; Homology models have been obtained by 3D-JIGSAW for the red parts of the sequences (Supplementary Figure S15).

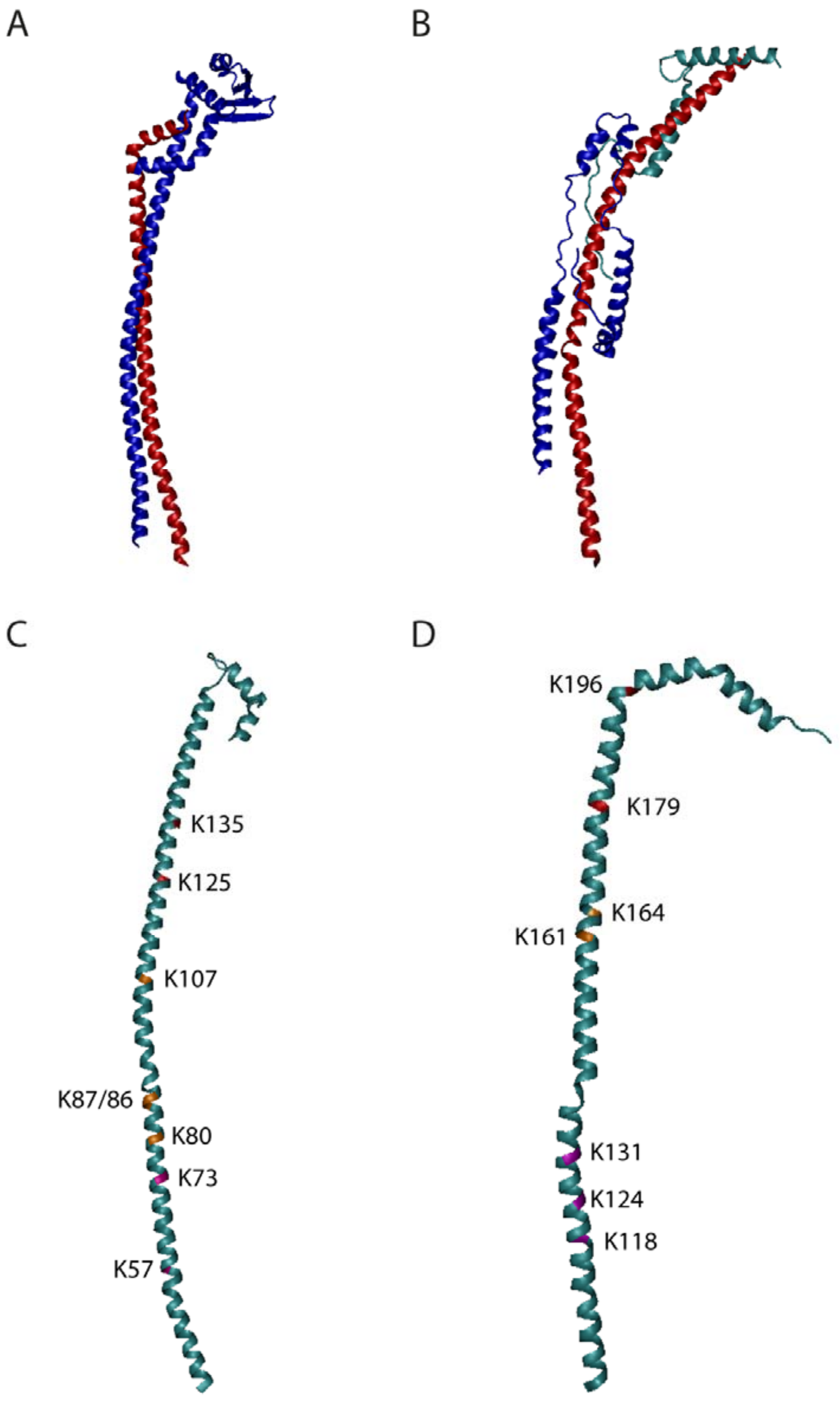

Supplementary Figure S15: Structures of the ATP synthase stators. (A) Crystal structure of the peripheral stalk of Thermus thermophilus H+-ATPase/synthase (pdb ID 3V6I). **(B)** Subcomplex of the stator of bovine mitochondrial ATP synthase (pdb ID 2CLY). **(C)** Homology model of subunit I obtained in this study. Intra-protein crosslinks are shown in red, orange and magenta. **(D)** Homology model of subunit II obtained in this study. Intra-protein crosslinks are shown in red, orange and magenta.

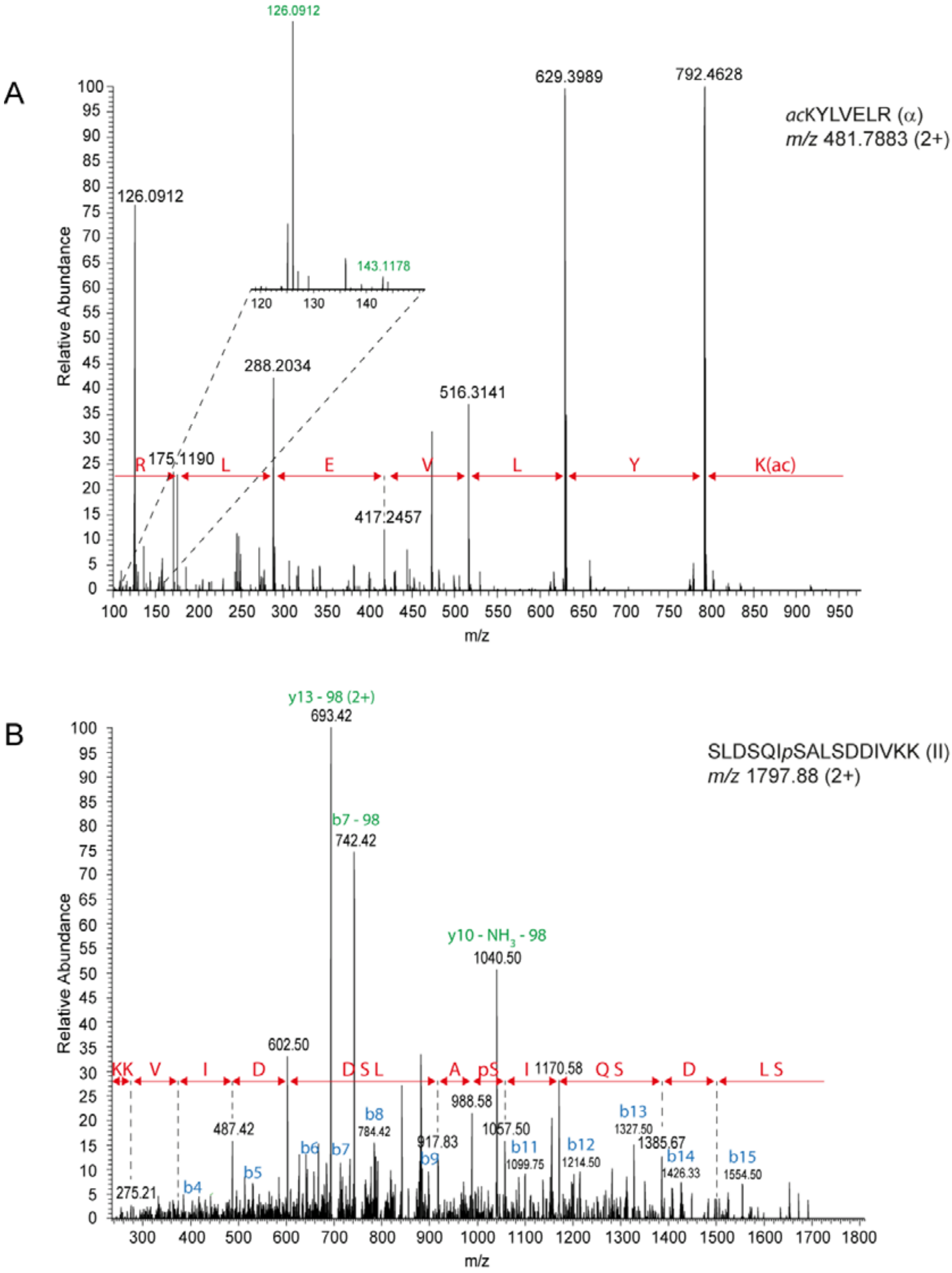

Supplementary Figure S16: Example spectra for identification of Lysine acetylation and phosphorylation. (A) HCD fragmentation delivers accurate masses of fragment and marker ions. The y-type ion series of the acetylated peptide *ac*KYLVELR (subunit α) is assigned in red. The magnification shows the marker ion region. Marker ions are labelled in green. In this example the mass deviation of the marker ions is 0.0001 amu. **(B)** MSMS spectrum of a phoophorylated peptide (SLSDQIpSALSDDIVKK, subunit II). The y-type ion series is assigned in red. B-type ions are labelled in blue and ions after loss of the phosphogroup (98 amu) are labelled in green.

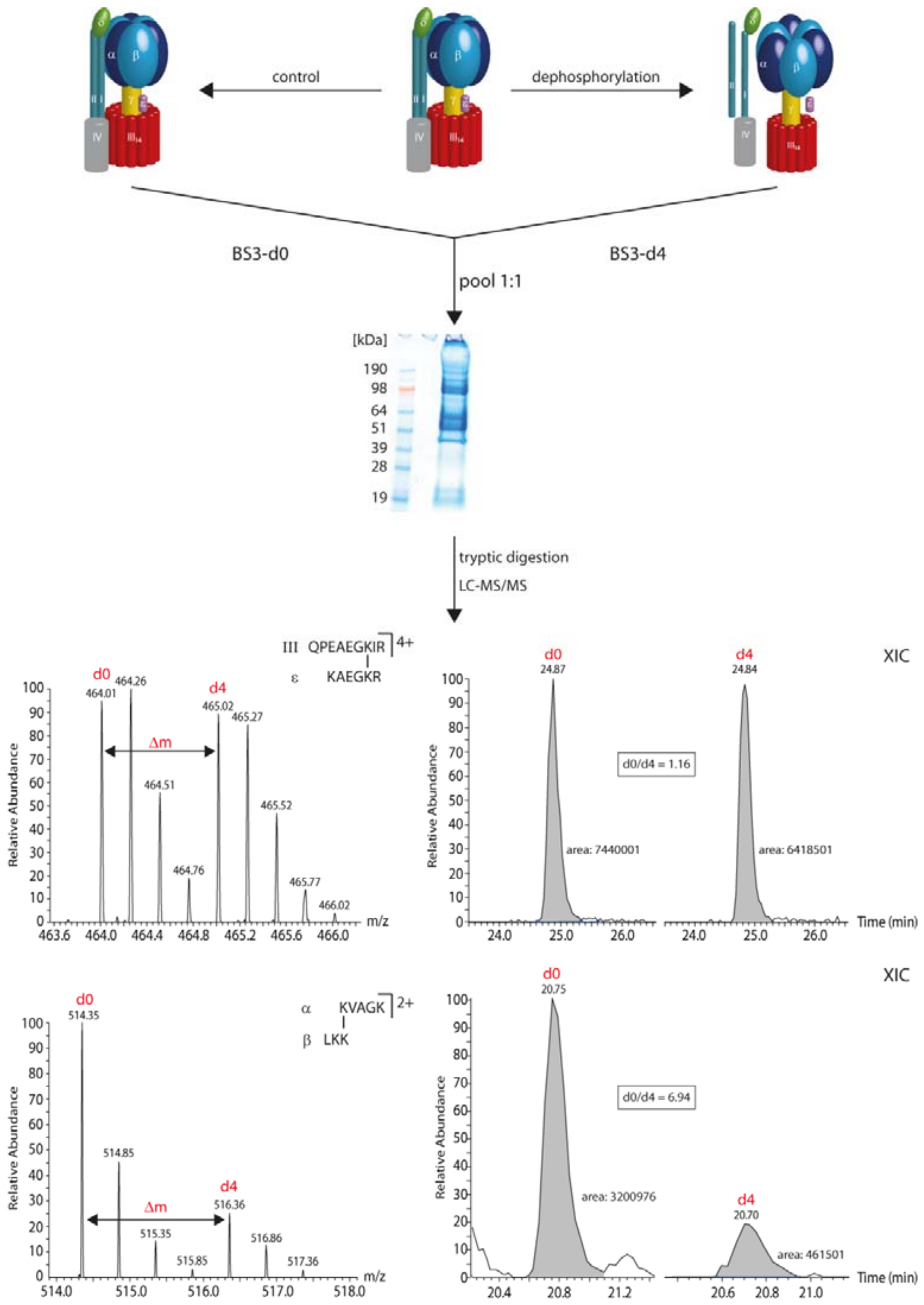

Supplementary Figure S17: Comparative crosslinking. The cATPase was dephosphorylated and crosslinked with deuterated BS3 (d4), while a control sample was crosslinked with non-deuterated BS3 (d0). The differently crosslinked samples were pooled in same amounts, proteins (protein complexes) were separated by SDS-PAGE and after tryptic digestion analysed by LC-MS/MS. Protein-protein interactions that were (partially) disrupted upon dephosphorylation of the cATPase show a decreased signal intensity in MS spectra for the deuterated crosslink. Extracted ion chromatograms (XICs) were generated for the deuterated and non-deuterated crosslinked peptide and quantitative ratios were obtained from the peak areas of the XICs. Two examples are shown: Interactions between ε and III are not affected by dephosphorylation (upper spectrum), whereas interactions between α and β were decreased (lower spectrum).

Supplementary Tables

Supplementary Table S1: Protein identification of cATPase protein subunits.

Protein	Accession no.	Mass [Da]	# MSMS spectra	# Peptide sequences	Sequence coverage [%]
cF_1 alpha	gi|216022791	55 417	687	41	50
cF_1 beta	gi|11497535	53 711	712	42	82
cF_1 gamma	gi|114643	40074	837	43	63
cF_1 delta	gi|114584	27 664	345	24	66
cF_1 epsilon	gi|11497534	14 691	442	20	100
cF_O I	gi|11497510	20 977	303	28	63
cF_O II	gi|461595	24 444	565	28	63
cF_O III	gi|237823447	7 592	7	4	56
cF_O IV	gi|11497512	27 070	19	5	37

The number of acquired MSMS spectra, determined peptide sequences and the sequence coverage are given for all 9 protein subunits. cF_O IV was identified with only low number of spectra and peptides and a low sequence coverage.

Supplementary Table S2: Accurate masses of cATPase protein subunits as determined by denaturing LC-MS.

Protein subunit	theoretical mass [Da]	experimental mass by LCMS [Da]	post-translationally processed
cF_1 α	55 451	55 353.31 ± 1.3	loss of N-terminal methionine, acetylation of N-terminus
cF_1 β	53 771	53 812.70 ± 2.5	N-terminal acetylation
cF_1 γ	40 074	35 818.23 ± 1.3	loss of N-terminal pre-sequence prior to import into chloroplasts [15]
cF_1 δ	27 681	20 484.92 ± 0.9	loss of N-terminal pre-sequence prior to import into chloroplasts [16]
cF_1 ε	14 700	14 568.03 ± 0.5	loss of N-terminal methionine
cF_O I	20 990	19 085.17 ± 0.5	N-terminal truncation [13]
cF_O II	24 460	16 497.44 ± 0.3	loss of N-terminal pre-sequence prior to import into chloroplasts [13, 16]
cF_O III	7 974	7 989.75 ± 0.3 - 8 050.01 ± 0.6	series of methionine oxidations
cF_O IV	27 087	25 122.21 ± 1.3	N-terminal truncation [17]

The theoretically available database masses as well as the experimentally determined masses are given for all proteins. Mass differences can be explained by the loss of N-terminal (pre-)sequences and by the loss of N-terminal methionine and/or acetylation of the N-terminus.

Supplementary Table S3: Lipids identified from the cATPase.

Lipid	m/z	XIC [area]	fatty acid side chains	
Sulfoquinovosyl diacylglycerol	815.48	1,366,497,486	18:3	16:0
	837.41	1,065,653,197	18:3	18:3
	817.5	455,814,625	18:2	16:0
	839.5	28,329,120	18:3	18:2
Glycosyldiacylglycerol	773.52	663,280,109	18:3	18:3
Diacylglycerophosphoglycerol	741.48	766,872,202	18:3	16:1
	747.5	349,478,474	18:1	16:0
	745.5	213,332,048	18:2	16:0
Diacylglycerophosphate	691.43	295,582,729	18:0	18:2
	693.45	106,405,678	18:3	18:2

Four different classes of lipids were identified. The m/z, the area as determined by extracted ion chromatograms (XIC) and the composition of the fatty acyl chains is given for all isomers.

Supplementary Table S4: cATPase complexes as determined by mass spectrometry.

Complex composition	Theoretical mass [kDa]	Theoretical mass [kDa] incl. ATP	Experimentally determined mass [kDa]	Mass error [Da]	Mass shift [Da]	Spectrum
$\alpha_3\ \beta_3\ \gamma\ \delta\ \varepsilon$ I II III_{14} IV	581.852	583.373	n/a	n/a	n/a	n/a
$\alpha_3\ \beta_3\ \gamma\ \ \varepsilon$ I II III_{14}	536.243	537.767	537.576	± 115	191	Figure 2
$\alpha_3\ \beta_3\ \gamma\ \ \varepsilon$ I II III_{14}	536.243	537.767	538.024	± 181	257	Figure 1
$\alpha_3\ \beta_3\ \gamma\ \delta\ \varepsilon$ III_{14}	521.372	522.893	523.281	± 155	399	Figure 1
$\gamma\ \ \varepsilon$ I II III_{14}	208.748	n/a	210.548	± 128	1800	Sup Figure 7
$\alpha_3\ \beta_3\ \gamma\ \delta\ \varepsilon$	398.365	399.889	400.451	± 177	562	Figure 2
$\alpha_3\ \beta_3\ \gamma\ \delta\ \varepsilon$	398.365	399.889	400.465	± 97	576	Figure 1
$\alpha_3\ \beta_3\ \gamma\ \delta\ \varepsilon$	398.365	399.889	400.581	± 72	692	Sup Figure 6
$\alpha_3\ \beta_3\ \gamma\ \delta$	383.797	385.321	385.932	± 27	611	Figure 2 - 1st CID
$\alpha_3\ \beta_3\ \gamma\ \delta$	383.797	385.321	385.949	± 42	628	Sup Figure 6
$\alpha_3\ \beta_3\ \gamma\ \delta$	383.797	385.321	385.966	± 19	645	Sup Figure 6 - 1st CID
$\alpha_3\ \beta_3\ \gamma\ \ \varepsilon$	377.881	379.405	379.976	± 186	571	Figure 2
$\alpha_3\ \beta_3\ \gamma\ \ \varepsilon$	377.881	379.405	380.087	± 84	682	Figure 2 - 1st CID
$\alpha_3\ \beta_3\ \gamma\ \ \varepsilon$	377.881	379.405	380.077	± 45	672	Sup Figure 6
$\alpha_3\ \beta_3\ \gamma\ \ \varepsilon$	377.881	379.405	380.066	± 55	661	Sup Figure 6 - 1st CID
$\alpha_3\ \beta_3\ \gamma$	363.313	364.837	365.448	± 47	611	Figure 2 - 1st CID
$\alpha_3\ \beta_3\ \gamma$	363.313	364.837	365.356	± 72	519	Figure 2 - 2nd CID
$\alpha_3\ \beta_3\ \gamma$	363.313	364.837	365.511	± 101	674	Sup Figure 6 - 1st CID
$\alpha_3\ \beta_3\ \gamma$	363.313	364.837	365.314	± 190	477	Sup Figure 6 - 2nd CID
$\alpha_3\ \beta_3\ \ \delta$	347.979	349.503	350.129	± 55	626	Sup Figure 6 - 2nd CID
$\alpha_3\ \beta_3\ \ \ \varepsilon$	342.063	343.587	344.319	± 44	732	Figure 2 - 2nd CID
$\alpha_3\ \beta_3$	327.495	329.019	329.573	± 54	554	Figure 2 - 2nd CID

The complex composition, the theoretical mass (including and excluding ATP), the experimentally determined mass, the mass error and the mass shift are given. All masses have been determined using software *Massign* [22]. Complexes from different spectra are listed.

*The theoretical mass has been calculated from accurate protein masses as determined in this study. It includes the lipid plug of 10.5 kDa for complexes that contain the membrane ring (III_{14}).
[#]Due to possible binding sites, 3ATP molecules have been accounted for all complexes.
[O]The mass shift is the mass difference between the theoretical and the experimental mass. It is caused by attachment of water and buffer molecules to the protein complex(es).

Supplementary Table S5: Crosslinking of the cATPase.

Protein 1	Protein 2	Peptide sequence 1	XL residue 1	Peptide sequence 2	XL residue 2	# spectra
alpha and beta						
alpha	alpha	ADEIS**K**IIR	11	**K**VAGK	378	1
alpha	alpha	VINALA**K**PIDGR	114	ADEIS**K**IIR	11	1
alpha	alpha	VGSAAQI**K**AMK	374	**K**VAGK	378	43
alpha		VGSAAQI**K**AM**K**K	374/377			62
alpha		AM**KK**VAGK	377/378			29
alpha		TYV**K**TN**K**PEFQEIISSTK	466/469			12
beta	beta	MPNIYNALIV**K**GR	50	**K**NLGR	18	53
beta		INPTTSDPGVSTLE**KK**NLGR	17/18			6
beta		GG**K**IGLFGGAGVG**K**TVLIMELINNIAK	167/178			4
beta		AMNLEMES**K**L**K**K	495/497			28
beta		AMNLEMES**K**LK**K**	495/498			12
alpha	beta	ADEIS**K**IIR	11	**K**NLGR	18	8
alpha	beta	VGSAAQI**K**AMK	374	**K**IER	426	10
alpha	beta	VGSAAQI**K**AMKK	374	LK**K**	498	1
alpha	beta	**K**VAGK	378	LK**K**	498	19
alpha	beta	VAG**K**LK	378	LK**K**	498	12
alpha	beta	TFTEEAEALL**K**EAIQEQMER	491	LK**K**	498	3
alpha	beta/I	**K**VAGK	378	L**K**K	497/86	2
delta						
delta	delta	LENDHLAQIA**K**GVQK	196	ITGA**K**NVR	205	2
delta		LENDHLAQIA**K**GVQ**K**ITGAK	196/200			22
delta		GVQ**K**ITGA**K**NVR	200/205			5
delta	alpha	LENDHLAQIA**K**GVQK	196	AM**K**K	377	2
delta	alpha	GVQ**K**ITGAK	200	ADEIS**K**IIR	11	1
delta	beta	ITGA**K**NVR	205	**K**NLGR	18	9
delta	beta	YGNEGSKLVDMSV**K**K	238	LK**K**	498	1
epsilon						
epsilon	epsilon	Q**K**IEANLALRR	112	**K**AEGK	105	1

epsilon	epsilon	Q**K**IEANLALRR	112	**K**AEGKR	105	5
epsilon	epsilon	Q**K**IEANLALRR	112	KAEG**K**R	109	1
epsilon	epsilon	RQ**K**IEANLALRR	112	**K**AEGK	105	1
epsilon	alpha	**K**AEGK	105	VGSAAQI**K**AMK	374	4
epsilon	beta	**K**AEGK	105	LK**K**	498	11
epsilon	beta	**K**AEGKR	105	LK**K**	498	17
epsilon	beta/I	**K**AEGK	105	L**K**K	497	2
epsilon	beta/I	**K**AEGKR	105	L**K**K	497	5
epsilon	beta/I	**K**AEGKRK	105	L**K**K	498	1
epsilon	gamma	KAEG**K**R	109	TV**K**K	116	1
epsilon	III	**K**AEGKR	105	QPEAEG**K**IR	48	4
epsilon	III	AEG**K**R	109	QPEAEG**K**IR	48	4
epsilon	III	KAEG**K**R	109	QPEAEG**K**IR	48	8
epsilon	III	Q**K**IEANLALR	112	QPEAEG**K**IR	48	4
epsilon	III	Q**K**IEANLALRR	112	QPEAEG**K**IR	48	2
gamma						
gamma	gamma	DRIGSV**K**NTQK	55	EG**K**LTVER	263	3
gamma	gamma	IGSV**K**NTQK	55	LVAAA**K**VR	71	1
gamma	gamma	IGSV**K**NTQK	55	**K**TLSINYNR	335	4
gamma	gamma	ITEAM**K**LVAAAK	65	IGSV**K**NTQK	55	2
gamma	gamma	LVAAA**K**VR	71	**K**GNTYFIR	163	1
gamma	gamma	GLCGGFNNMLL**K**K	139	**K**GNTYFIR	163	2
gamma	gamma	IAEL**K**K	149	TV**K**K	116	2
gamma	gamma	**K**LGVDYTIISIGK	150	**K**AESR	140	5
gamma	gamma	**K**LGVDYTIISIGKK	150	GLCGGFNNMLL**K**K	139	1
gamma	gamma	**K**LGVDYTIISIGKK	150	**K**AESR	140	3
gamma	gamma	KLGVDYTIISIG**K**K	162	GLCGGFNNMLL**K**K	139	1
gamma	gamma	LGVDYTIISIG**K**K	162	LVAAA**K**VR	71	1
gamma	gamma	FVSLV**K**SDPVIHTLLPLSPK	222	**K**AESR	140	1
gamma	gamma	GEICDING**K**CVDAAEDELFR	245	**K**AESR	140	1
gamma	gamma	MTAMSNATDNANEL**K**K	334	DRIGSV**K**NTQK	55	5
gamma	gamma	MTAMSNATDNANEL**K**K	334	IGSV**K**NTQK	55	16

gamma	gamma	FVSLV**K**SDPVIHTLLPLSPK	222	**K**AESR	140	1
gamma		IGSV**K**NTQ**K**ITEAMK	55/59			19
gamma		TV**KK**VALMVVTGDR	116/117			9
gamma		GLCGGFNNMLL**KK**AESR	139/140			11
gamma		IAEL**KK**LGVDYTIISIGK	149/150			20
gamma		LGVDYTIISIG**KK**GNTYFIR	162/163			2
gamma		LTT**K**EG**K**LTVER	260/263			19
gamma		MTAMSNATDNANEL**KK**TLSINYNR	334/335			1
gamma	beta	GLCGGFNNMLL**K**K	139	LK**K**	498	1
gamma	beta	**K**AESR	140	LK**K**	498	16
gamma	beta	**K**AESR	140	AMNLEMESKL**K**K	497	1
gamma	beta	**K**LGVDYTIISIGK	150	LK**K**	498	9
gamma	beta	**K**LGVDYTIISIGKK	150	AMNLEMESKLK**K**	498	1
gamma	beta	**K**LGVDYTIISIGKK	150	LK**K**	498	17
gamma	beta	**K**TLSINYNR	335	**K**IER	426	1
gamma	beta/I	**K**AESR	140	L**K**K	497/86	16
gamma	beta/I	**K**LGVDYTIISIGK	150	L**K**K	497/86	2
gamma	beta/I	FVSLV**K**SDPVIHTLLPLSPK	222	L**K**K	497	4
I and II						
I	I	G**K**AIEQLEK	73	**K**QR	57	1
I	I	AIEQLE**K**AR	80	**K**QR	57	1
I	I	L**K**KVEMDADQFR	86	AIEQLE**K**AR	80	2
I	I	**K**VEMDADQFR	87	AIEQLE**K**AR	80	17
I	I	LK**K**VEMDADQFR	87	AIEQLE**K**AR	80	4
I	I	E**K**MNLINSTYK	107	AIEQLE**K**AR	80	1
I		L**KK**VEMDADQFR	86/87			68
I		TLEQFENY**K**NETIQFEQQ**K**AINQVR	125/135			1
II	II	IYYTPLGDFMD**K**R	118	DASI**K**EQLSGVK	124	3
II	II	AEISAALN**K**MK	161	**K**ETQLEVEAK	164	7
II	II	IEVELQEALGSLEQQ**K**EDTIK	196	**K**K	179	3
II		DASI**K**EQLSGV**K**DTSSEVK	124/131			24
II		EQLSGV**K**DTSSEV**K**QLEEQANAVMR	131/138			2

II		AEISAALN**K**M**K**K	161/163			4
II		M**KK**ETQLEVEAK	163/164			35
II		**KK**IEVELQEALGSLEQQK	179/180			3
II	I	EQLSGV**K**DTSSEVK	131	G**K**AIEQLEK	73	6
II	I	EQLSGV**K**DTSSEVK	131	**K**VEMDADQFR	87	1
II	I	ETQLEVEA**K**LAEGR	173	E**K**MNLINSTYK	107	1
I	alpha	**K**QR	57	VGSAAQI**K**AMKK	374	2
I	alpha	**K**QR	57	VGSAAQIKAM**K**K	377	1
I	beta	AIEQLE**K**AR	80	LK**K**	498	8
I	beta	GKAIEQLE**K**AR	80	LK**K**	498	1
I	beta	**K**QR	57	L**K**K	497	1
I	beta/I	AIEQLE**K**AR	80	L**K**K	497/86	26
II	alpha	M**K**K	163	**K**YLVELR	456	12
II	alpha	M**K**K	163	TYV**K**TNKPEFQEIISSTK	466	3
II	alpha	**K**ETQLEVEAK	164	**K**YLVELR	456	5
II	alpha	SLDSQISALSDDIV**K**K	216	ADEIS**K**IIR	11	5
II	beta	**K**K	179	SAPAFTQLDT**K**LSIFETGIK	145	3

The two crosslinked proteins, the sequences of the crosslinked peptides, the crosslinked amino acid residues, and the number of identified MSMS spectra are given for every crosslink. Identified crosslinks are grouped according to their position and function within the cATPase. Intra-protein and intra-peptide crosslinks as well as inter-protein crosslinks are listed. Crosslinks with the peptide sequence LKK cannot unambiguously be assigned if the Lys in the middle is crosslinked. In this case crosslinks to β and I are possible. These crosslinks are listed with the inter-protein crosslinks in close proximity to the respective residues.

Supplementary Table S6: Homology modelling of γ, δ and ε using MODELLER web server.

Protein	gi-number	sequence length	modelled segment	template	template PDB ID	template region	MPQS
γ	114643	364	42-360	E. coli F1-ATP synthase	3OAA	1-283	1.21627
δ	114584	257	71-175	E.coli F-ATPase delta subunit N-terminal domain in complex with alpha subunit N-terminal 22 residues	2A7U	3-105	0.65856
ε	11497534	215	1-134	N-terminal beta-sandwich domain from T. Elongatus bp-1 f1; C-terminal alpha-helical domain from spinach chloroplast F1	2RQ7	1-134	1.42606

The gi-number, sequence length and modeled segment are given for the respective models. The corresponding template, its PDB ID as well as the template region are listed. Selection of the models has been performed on the basis of the MPQS (ModPipe Protein Quality Score). A model is considered a reliable model if the MPQS > 1.1 [23].

Supplementary Table S7: ATP synthase templates for homology modelling of the peripheral stalk subunits.

template	species	template pdb ID	I [SID]	II [SID]
Stator of bovine mitochondrial ATP synthase	*Bos taurus*	2CLY_A	0.06	0.02
Membrane domain of the subunit B of the E. coli ATP synthase	*E.coli*	1B9U_A	0.09	0.18
ATP synthase B subunit dimerization domain	*E.coli*	1L2P_A	0.14 / 0.15	0.15 / 0.25
NMR solution of the B30-82 domain of subunit B of E. coli F_1F_O ATP synthase	*E.coli*	2KHK_A	0.16	0.16
The domain features of the peripheral stalk subunit H of the M. jannaischii A1AO ATP synthase and the NMR solution structure H1-47	*M. jannaschii*	2K6I_A	0.15 / 0.2	0.19 / 0.10
NMR solution of the N-terminal domain of subunit E (E1-52) of A_1A_O ATP Synthase from M. Jannaischii	*M. jannaschii*	2KK7_A	0.11 / 0.14	0.18 / 0.14
Association of subuit D and E with G and the NMR solution structure of subunit G of the V_1V_O ATP synthase	*S. cerevisiae*	2K88_A	0.1 / 0.10	0.16 / 0.19
Peripheral stalk of Thermus thermophilus H^+-ATPase/Synthase	*T. thermophilus*	3V6I_A	0.11 / 0.06	0.13 / 0.24
Peripheral stalk of Thermus thermophilus H^+-ATPase/Synthase	*T. thermophilus*	3V6I_B	0.11 / 0.14	0.16 / 0.22

All templates have been utilized for both, subunit I and II. The sequence identities (SIDs) between the template fragments and the corresponding fragments within the sequence of I and II are listed. In most cases, several fragments have been used from the same template. The template description, species, pdb ID and the SIDs are given.

Supplementary Table S8: Summary of identified post-translational modifications of the cATase.

Protein	Peptide sequence	PTM	Peptide score	Marker ions*	Additional modifications
α	**K**YLVELR	Acetylation K456	27	126.0912; 143.1178	
	TN**K**PEFQEIISSTK	Trimethylation K393	32, 38	143.0811	
	GEITASE**S**R	Phosphorylation S127	24, 25, 31		
	TAVATDTILNQQGQNVICV**Y**VAIGQK	Phosphorylation Y196	24		
	VG**S**AAQIK	Phosphorylation S369	42		
	QPQSAPLTVEEQVMTIYTG**T**NGYLDSLELDQVR	Phosphorylation T442	15		
β	M**R**INPTTSDPGVSTLEK	Methylation R2	41, 43, 49, 55		Oxidation M1
	M**R**INPTTSDPGVSTLEKK	Methylation R2	32, 40, 44, 50, 54, 58		
	M**R**INPTTSDPGVSTLEKK	Trimethylation R2	40		
	V**K**ETLQR	Acetylation K392	34		
	D**T**AGQPMNVTCEVQQLLGNNRVR	Phosphorylation T54	39		Oxidation M59
	GRD**T**AGQP**M**NVTCEVQQLLGNNR	Phosphorylation T54	66, 72		Oxidation M59
	INPTT**S**DPGVSTLEK	Phosphorylation S8	51		
γ	**K**LGVDYTIISIGKK	Acetylation K150	74	126.0912; 143.1173	
δ	I**K**TVIDPSLVAGFTIR	Acetylation K210	47	126.0911	
	YASALADVADVTGTLEATN**S**DVEK	Phosphorylation S97	56, 57, 71		
ε	Q**K**IEANLALR	Acetylation K112	54	126.0541; 143.0934	
	**S**IWNSEVK	Phosphorylation S13	10, 16		
I	QRVFQQALQGALGTLNSCLNNELHL**R**	Methylation R167	26, 41		
	TLEQFENYKNETIQFEQQ**K**AINQV**R**	Methylation K135, R141	35		
	**K**VEMDADQFR	Acetylation K87			Oxidation M90
	TLEQFENYKNETIQFEQQ**K**	Acetylation K135	16, 21	143.119; 143.4911	
II	DASIKEQLSGV**K**DTSSEVK	Acetylation K131	22, 66		
	DA**S**IKEQLSGVKDTSSEVK	Phosphorylation S122	15, 30		
	SLDSQISAL**S**DDIVK	Phosphorylation S211	19, 63, 69, 73		

The peptide sequences, the PTMs and the modified amino acid residues, the Mascot peptide score, determined masses of marker ions and additional modifications, which can be caused during sample preparation, are given.

*Marker ions for Lysine acetylation: 143.1179 (IM), 126.0913 (IM-NH_3), 126.0919; Marker ions for Lysine trimethylation: 143.1543 (IM), 189.1598 (y_1): IM: immonium ion

Supplementary Table S9: Comparative crosslinking of the untreated and dephosphorylated cATPase.

Protein 1	Protein 2	Peptide sequence 1	XL residue 1	Peptide sequence 2	XL residue 2	# spectra	Fold change untr *vs* dephos
Inter-protein crosslinks							
alpha	beta	ADEIS**K**IIR	11	**K**NLGR	18	6	3.17
alpha	beta	VGSAAQI**K**AMK(K)	374	LK**K**	498	4	10.83
alpha	beta	AM**K**K	377	LK**K**	498	1	18.80
alpha	beta	**K**VAGK	378	AMNLEMES**K**LKK	495	2	3.81
alpha	beta	**K**VAGK	378	LK**K**	498	10	6.96
alpha	beta	VAG**K**LK	382	L**K**K	497	1	2.45
alpha	beta	VAG**K**LK	382	LK**K**	498	2	8.21
delta	beta	ITGA**K**NVR	205	**K**NLGR	18	2	3.86
I	alpha	**K**QR	57	VGSAAQIKAM**K**K	377	1	2.69
I	beta	**K**QR	57	**K**IER	426	1	2.71
I	beta	AIEQLE**K**AR	80	L**K**K	497	5	3.13
I	beta	AIEQLE**K**AR	80	LK**K**	498	4	2.20
I	beta	E**K**MNLINSTYK	107	LK**K**	498	1	2.14
II	alpha	M**K**K	163	VGSAAQIKAM**K**K	377	1	2.11
II	alpha	M**K**K	163	**K**YLVELR	456	5	4.35
II	alpha	M**K**K	163	TYV**K**TNKPEFQEIISSTK	466	1	2.87
II	I	DASIKEQLSGV**K**DTSSEVK	131	G**K**AIEQLEK	73	3	3.18
gamma	alpha	**K**AESR	140	**K**VAGK	378	1	2.13
gamma	beta	**K**AESR	140	AMNLEMES**K**LK	495	1	5.79
gamma	beta	**K**AESR	140	L**K**K	497	4	9.80
gamma	beta	**K**AESR	140	LK**K**	498	5	3.09
gamma	beta	**K**LGVDYTIISIGK	150	AMNLEMES**K**LKK	495	1	2.37
gamma	beta	**K**LGVDYTIISIGK	150	L**K**K	497	3	7.08
gamma	beta	**K**LGVDYTIISIGK	150	(AMNLEMESK)LK**K**	498	10	4.25
gamma	beta	FVSLV**K**SDPVIHTLLPLSPK	222	LK**K**	498	2	2.21
epsilon	beta	**K**AEGK	105	LK**K**	498	17	4.85
epsilon	beta	**K**AEGKR	105	L**K**K	497	3	4.35
epsilon	gamma	KAEG**K**R	109	TV**K**K	116	2	5.73

Intra-protein crosslinks							
alpha	alpha	EV**K**VVNTGTVLQVGDGIAR	25	ADEIS**K**IIR	11	4	0.58
alpha	alpha	VAG**K**LK	382	AM**K**K	377	79	1.21
alpha		TYV**K**TN**K**PEFQEIISSTK	466/469			24	1.01
beta	beta	GG**K**IGLFGGAGVGK	167	**K**IER	426	6	1.01
beta	beta	V**K**ETLQR	392	**K**IER	426	17	0.70
beta		AMNLEMES**K**L**K**K	495/497			91	1.29
delta		GVQ**K**ITGA**K**NVR	200/205			8	1.27
epsilon	epsilon	Q**K**IEANLALR	112	AEG**K**R	109	4	1.69
epsilon	epsilon	Q**K**IEANLALR	112	**K**AEGKR	105	12	1.16
I	I	AIEQLE**K**AR	80	**K**QR	57	13	0.99
II		AEISAALN**K**M**K**K	161/163			12	1.22

Inter-protein crosslinks that show different intensities in the untreated (untr) compared to the dephosphorylated (dephos) cATPase and some intra-protein crosslinks that show no differences are listed. The protein subunits, crosslinked peptide sequences and crosslinked amino acid residues, the number of MS/MS spectra as well as the fold change are given. A fold change > 1 represents crosslinks that are formed to a higher extent in the untreated cATPase.

Supplementary Methods

Purification of the intact chloroplast ATP synthase. 1 kg of spinach leaves was stored overnight at 4 °C in the dark to reduce starch content. The leaves were homogenized in 500 ml 0.4 M sucrose, 100 mM Tricine-NaOH pH 8.0, 2 mM $MgCl_2$, 1 mM PMSF. Complete Protease Inhibitor Tablets were added to the homogenization buffer. The homogenate was filtered through 4 layers of synthetic nylon net followed by centrifugation at 21.595 × g and 4 °C for 25 min. Pelleted chloroplasts and thylakoid membranes were resuspended in 500 ml 10 mM Tris-HCl pH 8.0, 0.5 mM $MgCl_2$, 0.1 mM PMSF and stirred on ice for 15 min. After centrifugation (see above) the pellet was washed with 0.4 M sucrose, 10 mM Tris-HCl pH 8.0, 150 mM NaCl, 0.5 mM $MgCl_2$, 0.1 mM PMSF. The pellet was frozen in liquid nitrogen and stored at -80 °C.

The pellet was resuspended in 0.4 M sucrose, 50 mM Tricine-NaOH pH 8.0, 2 mM $MgCl_2$ and the chlorophyll concentration was determined according to Wellburn and Lichtenthaler [50]. The concentration of chlorophyll was then adjusted to 5 mg/ml by addition of resuspension buffer. Solid DTT was added to a final concentration of 50 mM and the suspension was stirred on ice for 15 min. An equal volume of 20 mM Tricine-NaOH pH 8.0, 200 mM sucrose, 5 mM $MgCl_2$, 400 mM $(NH_4)_2SO_4$, 2 mM Na_2-ATP, 25 mM Na-cholate, 60 mM β-D-octylglucoside, 50 mM DTT was added and the suspension was stirred on ice for 30 min. Extracted proteins were separated by centrifugation at 218000 × g and 4 °C for 60 min. Extracted proteins were enriched in the supernatant by addition of saturated $(NH_4)_2SO_4$-solution to a final concentration of 1.2 M. After centrifugation at 10 000 × g and 4 °C for 15 min, cATPase proteins were precipitated by addition of saturated $(NH_4)_2SO_4$-solution to a final concentration of 1.8 M. Proteins were pelleted by centrifugation at 10 000 × g and 4 °C for 15 min. The pellet was resuspended in freezing buffer containing 30 mM NaH_2PO_4-NaOH pH 7.2, 200 mM sucrose, 2 mM $MgCl_2$, 0.5 mM Na_2-EDTA, 4 mM β-D-dodecylmaltoside (DDM) and frozen in liquid nitrogen.

The protein fractions from $(NH_4)_2SO_4$ precipitation were separated by sucrose density gradient centrifugation. A sucrose gradient from 12 to 30 % (w/v) sucrose was prepared in 30 mM NaH_2PO_4-NaOH pH 7.2, 2 mM $MgCl_2$, 0.5 mM Na_2-EDTA, 4 mM DDM and the sample was dissolved in the same buffer w/o sucrose. The protein sample was layered on top of the gradient and the complexes were separated at 182 000 × g and 4 °C for 22 h. Protein complexes were analyzed by SDS-PAGE and the band containing the ATP synthase was collected at approx. 24 % (w/v) sucrose. Intact ATP synthase complexes were separated by gel filtration on a Superdex 200 column at a flow rate of 0.5 ml/min using 30 mM NaH_2PO_4-NaOH pH 7.2, 2 mM $MgCl_2$, 0.5 mM Na_2-EDTA, 4 mM DDM buffer. 200 µl fractions were collected, frozen in liquid nitrogen and stored at -80 °C for MS analysis [40].

MS-based protein identification after in-gel digestion. cATPase proteins were digested with Trypsin in-gel after separation by SDS-PAGE [41]. For LC-MS/MS analysis, tryptic peptides were separated by nano-flow reversed-phase liquid chromatography (DionexUltiMate 3000 RSLC nano System, Thermo Scientific; mobile phase A, 0.1% (v/v) formic acid (FA); mobile phase B, 80 % (v/v) ACN/0.1% (v/v) FA) coupled to a LTQ-Orbitrap XL hybrid mass spectrometer (Thermo Scientific). The peptides were loaded onto a trap column (HPLC column Acclaim® PepMap 100, C18, 100 µm I.D. particle size 5µm; Thermo scientific) and separated with a flow rate of 300 nL/min on an analytical C18 capillary column (50 cm, HPLC column Acclaim® PepMap 100, C18, 75 µm I.D. particle size 3 µm; Thermo Scientific), with a gradient of 5-80 % (v/v) mobile phase B over 74 min. Peptides were directly eluted into the mass spectrometer.

Typical mass spectrometric conditions were: spray voltage of 1.8 kV; capillary temperature of 180 °C; normalized collision energy of 35 % at an activation of q = 0.25 and an activation time of 30 ms. The LTQ-Orbitrap XL was operated in data-dependent mode. Survey full scan MS spectra were acquired in the orbitrap (*m*/*z* 300−2000) with a resolution of 30,000 at *m*/*z* 400 and an automatic gain control (AGC) target at 10^6. The five most intense ions were selected for CID MS/MS fragmentation in the linear ion trap at an AGC target of 30,000. Detection in the linear ion trap of previously selected ions was dynamically excluded for 30 s. Singly charged ions as well as ions with unrecognized charge state were also excluded. Internal calibration of the orbitrap was performed using the lock mass option (lock mass: *m*/*z* 445.120025 [51]).

Raw data were searched against the NCBI non-redundant database (2011-06-24, 14478394 sequences) using the Mascot v2.3.02 search engine (Matrix Science). The mass accuracy filter was 10 ppm for precursor ions and 0.5 Da for MS/MS fragment ions. Peptides were defined to be tryptic with maximal two missed cleavage sites. Carbamidomethylation of cysteines and oxidation of methionine residues were allowed as variable modifications.

Lipid analysis. cATPase proteins were digested with Trypsin overnight at 37 °C and the peptide/lipid mixture generated was lyophilized and re-dissolved in 90 % (v/v) methanol/0.05 % (v/v) ammonia solution. For LC-MS/MS analysis, the peptide/lipid mixture was separated by nano-flow reversed-phase liquid chromatography (DionexUltiMate 3000 RSLC nano System, Thermo Scientific; mobile phase A, 70 % (v/v) methanol/0.05 % (v/v) ammonia solution; mobile phase B, 100 % (v/v) methanol/0.05 % (v/v) ammonia solution) coupled to a LTQ-Orbitrap XL hybrid mass spectrometer (Thermo Scientific). Peptides and lipids were loaded onto an analytical column (HPLC column Acclaim® PepMap 100, C18, 100 µm I.D. particle size 5µm; Thermo scientific) and separated with a flow rate of 300 nL/min and a gradient of 30-100 % (v/v) mobile phase B over 30 min.

Peptides/lipids were eluted directly into the mass spectrometer. Typical mass spectrometric conditions were: spray voltage of 1.5 kV and capillary temperature of 160 °C. The LTQ-Orbitrap XL was operated in negative ion mode and in data-dependent mode. Survey full scan MS spectra were acquired in the orbitrap (*m*/*z* 350−2000) with a resolution of 60,000 at *m*/*z* 400 and an automatic gain control (AGC) target at 5^5. The three most intense ions were selected for CID and HCD MS/MS fragmentation in the linear ion trap or the orbitrap, respectively. Detection in the linear ion trap and orbitrap of previously selected ions was dynamically excluded for 120 s. Ions with unrecognized charge state were excluded. The mass spectrometer was externally calibrated. CID fragmentation in the linear ion trap was performed at an AGC target of 30,000 and a normalized collision energy of 38 % at an activation of q = 0.25 and an activation time of 30 ms. HCD fragmentation was performed in the orbitrap with a resolution of 7500 at *m*/*z* 400 and a normalized collision energy of 40.0 % at an activation time of 40 ms.

Analysis of post-translational modifications. Proteins were digested in-gel as described. For detection of protein phosphorylation, phosphopeptides were enriched using titanium dioxide (TiO_2). Enrichment columns were packed in-house into pipette tips using TiO_2 material (GL Sciences, Japan). The columns were washed with 5 % (v/v) TFA/80 % (v/v) ACN and reconstituted in 20 % (m/v) 2,5-dihydroxybenzoic acid (DHB)/5 % (v/v) TFA/80 % (v/v) ACN. The peptides were dissolved in 20 % (m/v) DHB/5 % (v/v) TFA/80 % (v/v) ACN and loaded onto the material. After washing with 20 % (m/v) DHB/5 % (v/v) TFA/80 % (v/v) ACN and 5 % (v/v) TFA/80 % (v/v) ACN, phosphopeptides were eluted with 0.3 N ammonia solution (pH > 10.5). Eluted peptides were dried in a vacuum centrifuge for further MS analysis.

Tryptic peptides were separated by nano-flow reversed-phase liquid chromatography (HP 1100 series, Agilent; mobile phase A, 0.1% (v/v) formic acid (FA); mobile phase B, 95% (v/v) ACN/0.1% (v/v) FA) and subsequently analyzed on a LTQ-Orbitrap XL hybrid mass spectrometer (Thermo Scientific). The peptides were loaded onto a trap column packed in-house (2 cm, 360 µmo.d., 150 µmi.d.; ReproSil-Pur, C18, AQ 5 µm, Dr. Maisch HPLC GmbH) and separated with a flow rate of 300 nL/min on an analytical C18 capillary column packed in-house in a picofritcolumn (12 cm, 360 µm o.d., 75 µm i.d.; ReproSil-Pur, C18, AQ 3 µm, Dr. Maisch HPLC GmbH), with a gradient of 3-90 % (v/v) mobile phase B over 30 min.

CID fragmentation with multistage activation and HCD fragmentation were applied to identify peptide phosphorylation or acetylation, methylation and trimethylation, respectively. Typical mass spectrometric conditions for CID with multistage activation were: spray voltage of 1.8 kV; capillary temperature of 150 °C; normalized collision energy of 37.5 % at an activation of q = 0.25 and an activation time of 30ms. The LTQ-Orbitrap XL was operated in data-dependent mode. Survey full

scan MS spectra were acquired in the orbitrap (m/z 350−1600) with a resolution of 30,000 at m/z 400 and an automatic gain control (AGC) target at 10^6. The five most intense ions were selected for CID MS/MS fragmentation in the linear ion trap at an AGC target of 30,000. Multistage activation was enabled for neutral loss masses of one, two, three and four phosphosites per doubly and triply charged peptide. Detection in the linear ion trap of previously selected ions was dynamically excluded for 60 s. Singly charged ions as well as ions with unrecognized charge state were also excluded. Internal calibration of the orbitrap was performed using the lock mass option (lock mass: m/z 445.120025 [4]).

Typical mass spectrometric conditions for HCD fragmentation were: spray voltage of 1.8 kV; capillary temperature of 150 °C; normalized collision energy of 40.0 % at an activation time of 40 ms. The LTQ-Orbitrap was operated in data-dependent mode. Survey full scan MS spectra were acquired in the orbitrap (m/z 300−1400) with a resolution of 30,000 at m/z 400 and an automatic gain control (AGC) target at 10^6. The five most intense ions were selected for HCD MS/MS fragmentation in the HCD cell and detection in the orbitrap with a resolution of 7500 at m/z 400. Detection of previously selected ions was dynamically excluded for 30 s. Singly charged ions as well as ions with unrecognized charge states were also excluded. Internal calibration of the orbitrap was performed using the lock mass option (lock mass: m/z 445.120025 [51])

Proteins and their post-translational modifications were identified by database search using Mascot search engine (see above) including phosphorylation of serine, threonine and tyrosine residues as well as lysine acetylation as variable modifications.

Supplementary References

50. Wellburn, A.R. & Lichtenthaler, H. Formular and Program to Determine Total Carotenoids and Chlorophylls a and b of Leaf Extracts in Different Solvents, in *Advances in Photosynthesis Research*, C. Sybesma, Editor. The Hague, Boston, Lancaster. 9-12 (1984).
51. Olsen, J.V. *et al*. Parts per million mass accuracy on an Orbitrap mass spectrometer via lock mass injection into a C-trap. *Mol Cell Proteomics* **4**, 2010-2021 (2005).
